# An interspecific variation in rhizosphere effects on soil anti-erodibility

**DOI:** 10.1038/s41598-020-58784-z

**Published:** 2020-02-12

**Authors:** Zhenhong Wang, Alessandro Chiarucci, Hong Fang, Mouhui Chen

**Affiliations:** 1 Key Laboratory of Subsurface Hydrology and Ecological Effects in Arid Regions of the Ministry of Education, School of Water and Environment, Chang’an University, Xi’an, China; 2 Department of Biological, Geological and Environmental Science, University of Bologna, Via Irnerio, 42-40126 Bologna, Italy; 3 Water-affair Authority of Xifeng County, 551100 Guiyang, China

**Keywords:** Plant ecology, Ecology

## Abstract

Soil erosion due to underground leakage is a major factor causing land degradation in karst regions. Rhizosphere effects (REs) on soil anti-erodibility (SAE) can alleviate this type of soil erosion by improving soil physical processes such as aggregate stability. However, the magnitudes and causes of interspecific variation in REs on SAE remain unclear. We tested the rhizosphere SAE indices of 42 key woody species distributed worldwide. Biologically active matter (BAM) and analogs of antibiotics (AOAs) that affect the SAE in rhizosphere soils were tested by gas chromatography-mass spectrometry (GC-MS). We then used principal component analysis (PCA) and redundancy analysis (RA) to establish a spectrum of interspecific variability in the REs for the first time. The spectrum shows a gradient of change among species. Eleven species exerted negative REs on the SAE, while the remaining species showed positive effects along the spectrum. The species with large positive effects were mostly deciduous, which have high contents of both BAM and total organic matter and low contents of AOAs in their rhizosphere soil; compared with the other species tested, these species also have more leaves and roots and are better adapted to barren soils. The botanical characteristics of species with negative REs on the SAE differed from those with large positive effects. The contents of BAM in the rhizosphere accounted for 16–23% of the total variation in REs on the SAE. This study quantified interspecific variation in REs and identified root exudates with negative REs.

## Introduction

The rhizosphere consists of the soil surrounding plant roots and ranges from a fraction of a millimeter to approximately ten millimeters in thickness^1^. Rhizosphere effects (REs) are defined as biological, chemical, and physical changes in soils that occur because of root exudates and rhizodeposition^2,3^. Biological processes are related to the parasitism of host plant microorganisms that promote nutrient and water uptake from soils^2^. Moreover, plants and microorganisms release exudates, which improve the soil environment and provide food for animals and microbes in the soil^2,4,5^. The related chemical processes include primarily the impacts of exudate solutions on soil particles and rocks, the organic synthesis of exudates by plants and microbes, and the decomposition of organic matter induced by root exudates^6–8^. With respect to physical processes, polymer organics within root exudates can effectively bind soil particles together, promoting the formation of water-stable aggregates^9,10^. Microorganisms feeding on root exudates can also produce large quantities of hyphae and polysaccharides to bind soil particles together, leading to significant improvements to soil structure^11^. These two physical processes directly strengthen soil anti-erodibility (SAE) in the rhizosphere, which effectively provides resistance to soil erosion at the root-soil interface^12^.

REs on SAE greatly contribute to both the stability of the surface soil and the sustainability of the biosphere^12,13^. However, interspecific variation in the REs on SAE, i.e., the physical effects of the exudates, have not been largely investigated^14,15^. Globally, the REs on SAE are particularly highly important for the prevention of soil erosion caused by underground leakage in large karst regions^16^. Carbonate rocks are distributed worldwide and cover 5 × 10^7^ km^2^, which is equal to approximately 1/3 of the Earth’s surface. Among these areas, approximately 1.67 × 10^7^ km^2^ of carbonate rocks form outcrops on continents and shape a number of karst landscapes in Europe, the USA, Russia, and China (Supplementary Material S1). The primary type of soil erosion in karst landscapes is underground leakage. Soils are shallow and discontinuous in karst landscapes worldwide, but fissures, sinkholes, and underground drainage systems are highly prevalent^17–19^. When it rains, abundant underground runoff occurs with little superficial runoff and erosion, resulting in severe soil erosion via underground leakage. In fissures, soil is relatively rich, and plant growth therein is largely constrained. Plant roots release exudates within such fissures, having direct REs that in turn provide resistance to soil erosion via underground leakage and the preservation of carbon- and nutrient-rich soil^20,21^. Despite the key role of plants in such processes, knowledge of these aspects is only at a superficial level. The interspecific variation and magnitude and the cause of the REs on SAE for most globally distributed key woody plant species in karst regions need to be systematically investigated for effective application to the ecological engineering of soil erosion control.

Previous studies have investigated the direct REs on SAE, such as the adhesive properties of root mucilage and polysaccharides in the rhizosphere^22–24^, as well as the roles of biologically active matter (BAM) within the secretions of crop plants in the promotion of both hyphal growth and the release of polysaccharides from microbes to bind soil particles^25,26^. BAM comprises primarily total sugars, total amino acids, phenolic compounds, and free amino acids^27–29^. BAM is a chemical precursor for the biological synthesis of polysaccharides, proteins and root mucilage and can bind soil particles with its adhesive properties^27–29^. In addition, root exudates contain not only a variety of different types of mucilage, polysaccharides and BAM but also some analogs of antibiotics (AOAs), such as amides, phenolic ethers, aldehydes, and ketones^30,31^. These types of organic matter are the chemical AOAs or precursors used to synthesize antibiotics^27–29^. From a physiological perspective, these AOAs may limit the growth of microbes by disrupting metabolism because their chemical structure is very similar to that of antibiotics, resulting in negative REs, but no studies have tested these effects. Moreover, all of these studies discussed above focused on a few crop species, such as wheat, soybean and corn, from which it is easy to collect rhizosphere soil and root exudates and quantify REs^1,32^. Many karst regions are covered by natural and seminatural vegetation consisting of different woody plant species that form deep root systems. These woody plant species have a major role in structuring terrestrial ecosystems, but it is not easy to investigate their REs because the soil samples must be collected from root systems within narrow fissures among rocks in karst habitats. In addition, the determination of organic matter within the exudates of rhizosphere soil via gas chromatography-mass spectrometry (GC-MS) involves many complex steps^33^. Therefore, studies on interspecific variation in and causes of the REs of woody plant species are challenging^34^.

In this study, we aimed specifically to investigate the interspecific variation and corresponding causes of REs on the SAE properties of globally distributed major woody plant species in one of the major karst regions of the world, the Guizhou karst region, China^17,21^. We assumed that there were significant differences in the REs on SAE among the 42 species, and interspecific variation partially resulted from the different secretions of BAM and AOAs from these plant species, which promoted a series of processes such as the adhesion of soil particles and the growth of microbes to bind soil particles^35,36^. First, we identified the major species and collected soil samples from the rhizospheres of those species. Second, the BAM and AOA concentrations and seven SAE indices of the soil samples were tested. Third, we established a spectrum that integrated the seven SAE indices to describe the magnitude of the interspecific variation in the REs on SAE on the basis of principal component analysis (PCA) and redundancy analysis (RA). Fourth, regression analysis was conducted to reveal the relationships between the contents of BAM and AOAs from different species and the REs on SAE. We address the following questions: (1) How much of the REs on SAE described by the seven SAE indices varies among key species? (2) Can we establish a spectrum via a suitable method to combine the seven indices and quantify the interspecific variation in the REs on the SAE? What information related to erosion control can be indicated by the spectrum? (3) Are BAM and AOAs factors that cause interspecific variation in the REs on SAE?

## Results

### Rhizosphere and non-rhizosphere SAE

Of the seven measured SAE indices, three (aggregation status, particle fractal dimension and organic matter content) presented significantly higher values in the rhizosphere zones than in the non-rhizosphere zones (Table 1), while two (the dispersion ratio and dispersion coefficient) presented significantly lower values. The degree of aggregation and microaggregate fractal dimension did not significantly differ (Table 1). Notably, the particle and microaggregate fractal dimensions of the rhizosphere zones were larger than those of the non-rhizosphere zones. The two SAE indices were calculated as *d*_i_ = 0.25 mm and *d*_i+1_ = *d*_max_ = 2 mm. The rhizosphere soils contained many particles with a *d*_i_ larger than 0.25, 0.05, or 0.02 mm in their mechanical components (not indicated), which was possibly the result of the corrosive effects of root exudates.

**Table 1:** SAE indices of the rhizosphere (R) and non-rhizosphere (N) zones.

SAE indices	Position	Average (%)	Sampling number	Standard deviation	t	P
Aggregation status	R	51.17	126	14.47	3.014	0.0044 < 0.01
	N	48.04	126	14.47		
Degree of aggregation	R	70.61	126	15.84	1.484	0.1455 > 0.05
	N	69.04	126	16.25		
Dispersion ratio	R	36.29	126	13.25	−3.343	0.0018 < 0.01
	N	40.09	126	13.23		
Dispersion coefficient	R	25.06	126	10.04	−2.024	0.04957 < 0.05
	N	28.12	126	12.95		
Particle fractal dimension	R	2.77	126	0.06	2.94	0.0050 < 0.01
	N	2.75	126	0.07		
Microaggregate fractal dimension	R	2.57	126	0.06	0.94	0.3529 > 0.05
	N	2.56	126	0.06		
Organic matter content	R	112.15	126	0.01	4.30	0.00004 < 0.01
	N	87.09				

Student’s t-tests were applied to verify the significance of differences between R and N.

### REs of each species on SAE

Of the 42 investigated species, 29 presented positive values for aggregation status, while 13 species presented negative values, indicating positive and negative REs, respectively (Table 2). Similarly, plant species with a negative aggregation status also presented negative degree of aggregations. Except for that of two species, organic matter presented positive values, but the dispersion ratio and coefficients were mostly negative. All of the particle and microaggregate fractal dimensions were very small, and nearly half of the values were negative. These negative dispersion ratios, dispersion coefficients, and particle and microaggregate fractal dimensions indicated positive effects on the SAE. The interspecific variation in the REs was evidently significant according to the statistical quantities, i.e., the coefficient of variation (CV) corresponding to each index (Table 2). Unexpectedly, the differences in all of these indices except for the degree of aggregation were not significant between the 22 evergreen and 20 deciduous plant species (Table 2).

**Table 2:** The REs of plants on SAE.

Species name	Aggregation status, x_1_ (%)	Degree of aggregation, x_2_ (%)	Dispersion ratio, x_3_ (%)	Dispersion coefficient, x_4_ (%)	Organic matter content, x_5_ (g/kg)	Particle fractal dimension, x_6_	Microaggregate fractal dimension, x_7_
*Itea yunnanensis*	5.28 ± 0.15	5.76 ± 0.14	−3.02 ± 0.08	0.88 ± 0.03	3.44 ± 0.10	0.002 ± 0.000	0.001 ± 0.000
*Quercus aliena*	2.42 ± 0.10	−2.41 ± 0.09	−4.61 ± 0.11	−1.10 ± 0.05	13.63 ± 0.50	−0.032 ± 0.001	−0.036 ± 0.001
*Ligustrum lucidum*	6.59 ± 0.21	−3.82 ± 0.12	−10.94 ± 0.31	−45.24 ± 1.88	41.62 ± 2.15	0.025 ± 0.001	−0.095 ± 0.003
*Ilex chinensis*	3.85 ± 0.08	2.59 ± 0.12	−3.17 ± 0.10	−2.45 ± 0.09	67.38 ± 3.12	0.032 ± 0.001	0.020 ± 0.000
*Cinnamomum glanduliferum*	−13.09 ± 0.37	−3.64 ± 0.08	14.61 ± 1.08	18.34 ± 0.75	1.03 ± 0.04	0.029 ± 0.001	0.086 ± 0.003
*Albizia kalkora* (Roxb.) Prain	−3.32 + 0.10	−8.45 ± 0.31	−3.25 ± 0.14	−4.65 ± 0.11	17.56 ± 0.60	0.030 ± 0.001	−0.020 ± 0.000
*Celtis sinensis*	−2.28 ± 0.06	−2.79 ± 0.09	0.47 ± 0.03	1.40 ± 0.06	35.76 ± 1.09	−0.022 ± 0.001	0.009 ± 0.000
*Cudrania tricuspidata*	1.63 ± 0.06	2.66 ± 0.10	0.05 ± 0.003	−7.24 ± 0.29	10.16 ± 0.47	0.025 ± 0.001	−0.048 ± 0.002
*Broussonetia papyrifera*	3.82 ± 0.09	5.93 ± 0.17	−2.29 ± 0.09	−17.24 ± 0.69	8.88 ± 0.35	0.035 ± 0.001	−0.040 ± 0.001
*Zanthoxylum planispinum* Sieb. et Zucc.	6.59 ± 0.28	0.75 ± 0.07	−8.49 ± 0.28	22.07 ± 0.91	−6.57 ± 0.25	0.004 ± 0.000	−0.103 ± 0.004
*Toddalia asiatica*	1.20 ± 0.03	3.64 ± 0.10	1.06 ± 0.05	−6.77 ± 0.23	18.48 ± 0.83	−0.025 ± 0.001	−0.059 ± 0.002
*Rhamnella martinii*	7.00 ± 0.17	0.37 ± 0.04	−9.34 ± 0.33	−10.02 ± 0.39	22.69 ± 0.82	0.013 ± 0.000	−0.029 ± 0.001
*Diospyros kaki* var. silvestris	−0.95 ± 0.02	−4.72 ± 0.15	−2.28 ± 0.08	1.18 ± 0.04	50.69 ± 1.77	−0.025 ± 0.001	−0.003 ± 0.000
*Rhus chinensis*	12.24 ± 0.48	8.65 ± 0.30	−9.33 ± 0.27	18.21 ± 0.88	2.84 ± 0.11	0.022 ± 0.001	0.188 ± 0.007
*Ilex corallina*	−0.39 ± 0.01	−1.30 ± 0.08	−0.76 ± 0.02	−3.52 ± 0.12	25.43 ± 1.01	−0.062 ± 0.002	0.022 ± 0.001
*Catalpa fargesii* f. duclouxii	5.52 ± 0.11	1.00 ± 0.02	−6.27 ± 0.17	−1.15 ± 0.03	26.32 ± 1.28	−0.010 ± 0.000	−0.003 ± 0.001
*Diospyros cathayensis*	3.40 ± 0.11	2.83 ± 0.11	−1.98 ± 0.08	8.95 ± 0.44	14.50 ± 0.45	−0.011 ± 0.000	0.041 ± 0.002
*Viburnum chinshanense*	15.37 ± 0.90	19.61 ± 1.08	−9.54 ± 0.32	−7.56 ± 0.36	27.05 ± 1.39	−0.036 ± 0.001	−0.046 ± 0.002
*Elaeagnus pungens*	−1.32 ± 0.02	−5.12 ± 0.22	−0.98 ± 0.03	−0.89 ± 0.03	41.64 ± 1.77	0.016 ± 0.000	0.004 ± 0.000
*Lindera communis*	4.15 ± 0.09	2.00 ± 0.08	−4.32 ± 0.14	−16.40 ± 0.55	25.28 ± 1.31	0.137 ± 0.006	0.049 ± 0.002
*Pyracantha atalantioides*	9.81 ± 0.22	8.49 ± 0.35	−9.53 ± 0.25	−11.32 ± 0.50	19.83 ± 1.80	−0.006 ± 0.000	−0.072 ± 0.003
*Litsea cubeba*	−2.78 ± 0.07	−5.43 ± 0.17	2.03 ± 0.07	−23.15 ± 1.04	17.03 ± 1.70	0.039 ± 0.001	−0.038 ± 0.002
*Machilus microcarpa*	24.08 ± 1.50	11.01 ± 0.28	−32.05 ± 1.66	7.01 ± 0.26	54.13 ± 2.03	−0.003 ± 0.000	0.064 ± 0.002
*Ilex chinensis* Hook. et Arn. var. oblonga	1.81 ± 0.05	3.91 ± 0.20	−1.21 ± 0.10	−12.17 ± 0.54	9.36 ± 0.38	0.030 ± 0.001	−0.030 ± 0.001
*Ilex memecylifolia*	−0.21 ± 0.01	−2.09 ± 0.09	−1.33 ± 0.05	−1.51 ± 0.06	6.32 ± 0.25	0.008 ± 0.000	0.006 ± 0.000
*Liquidambar formosana*	−6.28 ± 0.15	−7.19 ± 0.32	8.03 ± 0.22	−2.19 ± 0.09	26.99 ± 1.08	0.020 ± 0.001	0.026 ± 0.001
*Ligustrum sinense*	−3.02 ± 0.07	0.11 ± 0.01	6.22 ± 0.23	−0.77 ± 0.03	36.16 ± 1.49	0.031 ± 0.001	0.022 ± 0.001
*Mallotus philippensis*	−6.95 ± 0.19	−5.22 ± 0.12	9.04 ± 0.35	−9.04 ± 0.37	25.56 ± 1.06	0.039 ± 0.001	−0.007 ± 0.000
*Lithocarpus glaber* (Thunb.) Nakai	3.01 ± 0.07	0.96 ± 0.07	−8.53 ± 0.39	−1.97 ± 0.08	9.61 ± 0.42	0.015 ± 0.000	0.008 ± 0.000
*Armeniaca mume*	17.01 ± 0.84	24.02 ± 1.89	−15.86 ± 0.40	11.96 ± 0.48	38.20 ± 1.87	−0.057 ± 0.002	−0.006 ± 0.000
*Clerodendrum mandarinorum*	0.05 ± 0.00	−1.50 + 0.12	−2.15 ± 0.03	4.04 ± 0.16	−19.45 ± 0.98	−0.017 ± 0.000	0.006 ± 0.000
*Cyclobalanopsis glauca*	8.41 ± 0.24	−8.00 ± 0.29	−12.53 ± 0.36	−7.00 ± 0.29	6.60 ± 0.27	0.008 ± 0.000	−0.022 ± 0.001
*Evodia trichotoma* (Lour.) Pierre	−5.86 ± 0.12	−10.64 ± 0.15	−1.20 ± 0.04	−13.10 ± 0.55	39.98 ± 1.80	0.027 ± 0.001	−0.037 ± 0.001
*Lithocarpus confinis*	5.95 ± 0.14	−9.03 ± 0.29	−2.27 ± 0.09	−4.00 ± 0.11	21.92 ± 0.90	0.051 ± 0.002	0.036 ± 0.001
*Swida wilsoniana*	3.84 ± 0.08	4.99 ± 0.15	−1.40 ± ± 0.02	22.93 ± 1.03	28.03 ± 1.63	0.104 ± 0.004	0.031 ± 0.001
*Evodia fargesii* Dode	6.77 ± 0.17	1.88 ± 0.05	−7.33 ± 0.24	−2.09 ± 0.08	46.97 ± 2.05	−0.007 ± 0.000	−0.019 ± 0.000
*Vitex canescens*	−4.60 ± 0.13	−7.16 ± 0.31	0.18 ± 0.01	−0.25 ± 0.02	58.01 ± 2.55	−0.011 ± 0.000	−0.011 ± 0.000
*Carpinus pubescens*	3.17 ± 0.08	−1.30 ± 0.02	−5.50 ± 0.25	−2.19 ± 0.12	41.71 ± 1.57	0.043 ± 0.002	−0.011 ± 0.001
*Nothopanax davidii*	3.62 ± 0.07	2.38 ± 0.11	−3.58 ± 0.12	7.43 ± 0.32	36.31 ± 2.10	−0.017 ± 0.000	0.122 ± 0.005
*Platycarya longipes*	5.29 ± 0.11	−1.50 ± 0.07	−7.99 ± 0.28	−3.13 ± 0.21	49.85 ± 2.08	0.038 ± 0.002	0.024 ± 0.001
*Cyclobalanopsis gracilis*	7.99 ± 0.19	6.23 ± 0.22	−7.51 ± 0.30	−7.97 ± 0.42	8.60 ± 0.39	0.042 ± 0.001	0.018 ± 0.000
*Cladrastis platycarpa*	0.45 ± 0.02	2.92 ± 0.04	1.65 ± 0.07	1.93 ± 0.11	43.01 ± 1.69	0.044 ± 0.001	0.070 ± 0.003
Maximum (%)	24.08	24.02	14.61	22.93	67.38	0.14	0.19
Minimum (%)	−13.09	−10.64	−32.05	−45.24	−19.45	−0.06	−0.10
Mean (*μ, %*)	3.08	1.56	−3.74	−3.47	25.06	0.02	0.008
Variance (*σ*)	45.39	46.98	54.46	138.16	348.78	0.00	0.003
Standard deviation	6.74	6.85	7.38	11.75	18.68	0.04	0.053
CV (%)^a^	218.87	439.88	197.23	339.07	74.52	218.58	689.71
N	42	42	42	42	42	42	42
F-value^b^	0.93	3.51	0.44	0.58	0.54	1.10	1.02
*p*	0.87	0.01	0.07	0.23	0.18	0.83	0.97

^a^CV (%) = Standard deviation × 100/mean; ^b^The F-value and p are the results according to one-way ANOVA and the Duncan multiple range test, respectively (at a confidence level of 95%), on both groups of plants, i.e., 22 evergreen and 20 deciduous plant species.

### Contents of BAM and AOAs

**Figure 1:**
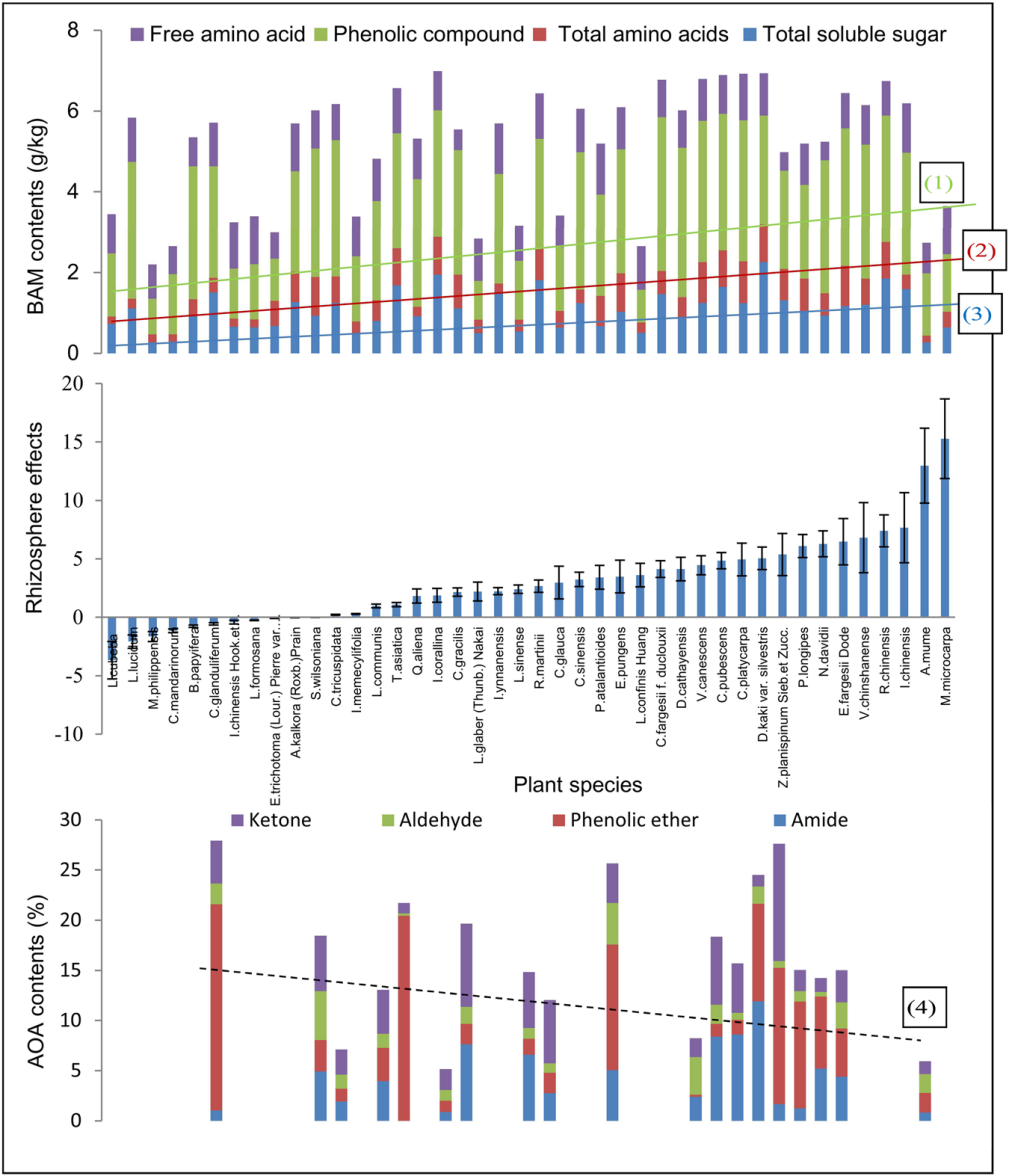
Spectrum of the REs according to PCA for 42 woody species. The spectrum was also linked to the different contents of BAM and AOAs in the rhizosphere of each species. The trend lines show changes in the contents of BAM and AOAs corresponding to the spectra of the REs for these tree species: (1) phenolic compounds, y = 0.0205x + 2.0825, R2 = 0.4370, p < 0.01; (2) total amino acid, y = 0.0093x + 0.3637, R2 = 0.6574, p < 0.01; (3) total sugars, y = 0.0109x + 0.82515, R2 = 0.4785, p < 0.05; (4) phenolic ethers, y = −0.01104x + 8.9335, R2 = 0.5289, p < 0.05. The trend lines for free amino acids, ketones, aldehydes and amides are not statistically significant, so they were excluded from the figure. The three small figures have a common axis showing the name of the plant species. The same scheme applies below.

Among the contents of the four types of BAM, the content of the phenolic compounds was highest, whereas that of the free amino acids was the lowest (Fig. 1). The statistical summaries revealed great interspecific variation (Supplementary Material S2). Among the relative content of the four AOAs, that of the phenolic ethers was the highest, whereas that of the aldehydes was the lowest (Fig. 1). The corresponding statistical summaries also revealed great interspecific variation in the AOA content.

### Spectrum based on PCA

The spectrum showed different effects in the rhizosphere from each species (Fig. 1). As shown at the right side of the spectrum, eleven species (*M. microcarpa*, *A. mume*, *I. chinensis*, *R. chinensis*, *V. chinshanense*, *E. fargesii* Dode, *N. davidii*, *P. longipes*, *Z. planispinum* Sieb. et Zucc., *D. kaki* var. silvestris and *C. platycarpa*) had the largest effects on SAE. These eleven species with the largest effects (with the notable exceptions of *M. microcarpa* and *A. mume*) presented high contents of total sugars, total amino acids and phenolic compounds in their rhizospheres. However, the other eleven species at the left side of the spectrum showed negative effects. With the exception of five species that presented high contents of phenolic compounds, the eleven negative-effect species evidently presented low contents of total sugars, total amino acids and phenolic compounds in their rhizospheres. The contents of the three BAM types exhibited an obviously increasing trend from left to right. The AOA contents were irregular; however, the AOA contents decreased to varying degrees along the spectrum from left to right if the high-content AOA species *D. kaki* var. silvestris and *C. platycarpa* were not considered at the right of the spectrum.

### Spectrum based on RA

**Figure 2:**
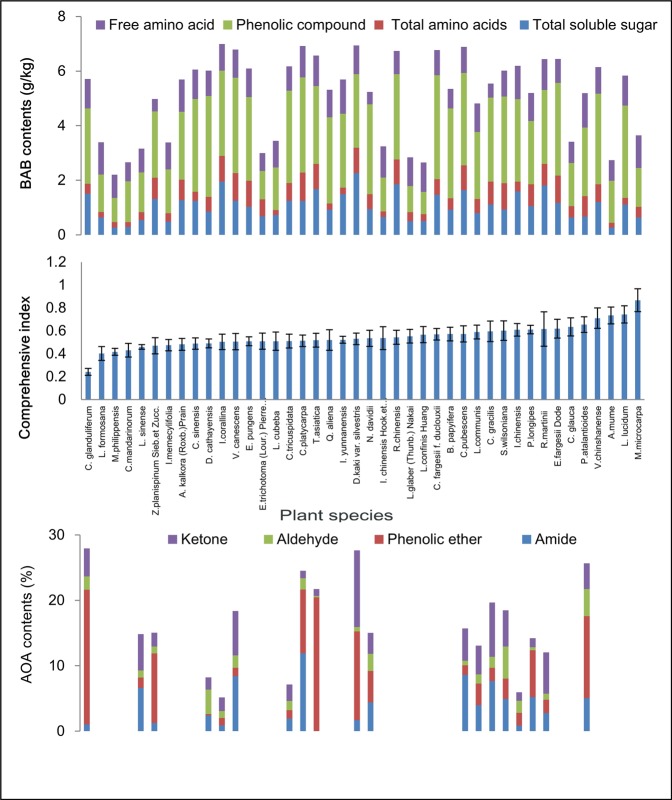
Spectrum of the REs according to RA for 42 woody species. All the trend lines that show changes in the contents of BAM and AOAs corresponding to the spectra of the REs for these tree species are not statistically significant, so were excluded from the figure.

The spectrum also showed the size of the REs (Fig. 2). On the right, eleven species, *A. mume*, *V. chinshanense*, *P. longipes*, *N.* *davidii*, *D. kaki var. silvestris*, *R. chinensis*, *L. sinense*, *M. microcarpa*, *L. communis*, *C.* *gracilis*, and *E. fargesii Dode*, had larger effects than did the other species. Conversely, the other eleven species at the left side of the spectrum exerted relatively small effects. However, the BAM and AOA contents changed irregularly along the spectrum.

### Relationships between the comprehensive index and BAM and AOAs

**Figure 3:**
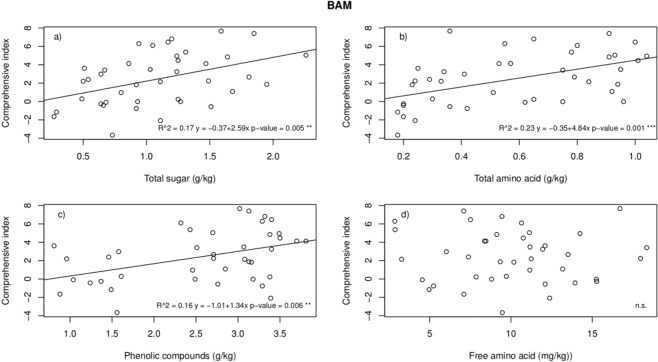
Regression analysis between the comprehensive index of the REs and the contents of BAM. The asterisks (*) represent significance at the 95% confidence level.

There were significant positive relationships between the comprehensive index of SAE and the total sugar, total amino acid, and phenolic compound contents in the rhizospheres of the 42 species (Fig. 3). The results indicated that the REs of these species strengthened with increasing BAM contents regardless of whether the BAM originated from the roots of these plants, from microbes or from other processes in the rhizosphere. Three types of BAM explained 16–23% of the total variation in the REs (Fig. 3A–C). However, the content of free amino acids was not significantly related to the index (Fig. 3D), although this content may be too low to play a substantial role. The results explained the trend of the spectrum when the BAM increased (Fig. 1).

**Figure 4:**
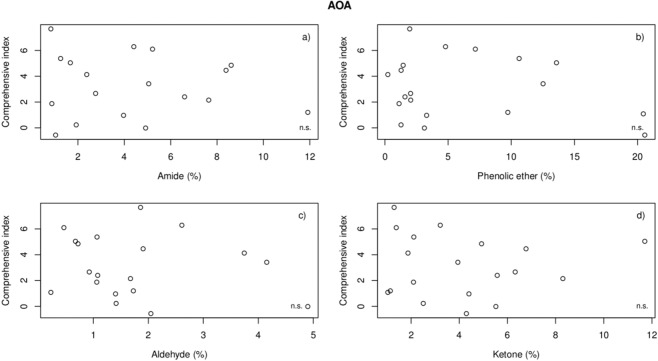
Regression analysis between the comprehensive index of the REs and the contents of AOAs.

There were no significant relationships between the comprehensive index and the contents of the four AOAs in the rhizospheres of the 42 species (Fig. 4). However, the index tended to decrease with increasing AOA content, which validated the negative effects of AOAs on the REs, which is in line with previous assumptions. The results of the statistical analysis were identical to the results shown in Fig. 2.

## Discussion

### Variation in the REs on SAE according to seven indices

On the basis of a test of seven SAE indices of the rhizosphere and non-rhizosphere zones, the average REs of 42 key woody species on the SAE were small. These results were not as impactful as expected when they were compared with the results obtained from rhizosphere soils of wheat, soybean and corn^1,32^. However, the variation in these indices among the investigated species was as obvious as that of other REs related to biological and chemical processes in previous studies^2^. This indicates that some plant species strongly contribute to the REs on SAE. The size of the contribution is dependent on the species investigated. Specifically, more than half of the studied species contributed largely to the REs on the SAE, while some plants exhibited a negative contribution. These results were not in agreement with the consistently positive effects described in previous studies^1^, in which a few crop species were investigated but no negative REs on SAE were detected. The negative effects in the present study occurred when the test values of the SAE indices in the non-rhizosphere zone of the plant species were larger than those in the rhizosphere zone of the same species. This occurred primarily because there was a higher percentage of fine soil particles (diameter <0.05 mm), possibly caused by the corrosive effects of root exudates, in the rhizosphere zone of the species with negative effects than in the non-rhizosphere zone. Because there were negative and positive effects of these 42 woody plant species with respect to their REs on SAE, the interspecific variation in REs on SAE were more significant for these plant species than for plant species in previous studies^1,2,32^. These great interspecific variation in the REs on SAE indicate that plant species play a different role in the maintenance of soil stability and affect soil conservation and soil nutrient levels, which conversely support the growth and adaptation of these plants to barren soil in fragile karst regions. These findings add new knowledge concerning the effects of vegetation on SAE and clarify the roles of plant species in complex plant-soil systems.

### Spectra and their indicated information

The REs on SAE were quantified via seven indices, and this traditional method demonstrated different measures of the REs on the SAE. However, this method resulted in some scattered information to describe the REs on SAE incompletely^37^. Therefore, we used a comprehensive index derived from PCA and RA and established two respective spectra of the comprehensive REs on SAE for the 42 woody plant species to quantify the REs of each species on the SAE. The REs of these species presented by the two spectra were largely similar on the basis of their validation of each other. Therefore, the two-spectrum method can be used to quantify the REs of different species on SAE and to determine the plant species with high REs on SAE for soil erosion control applications. However, there were also differences between the two spectra because the use of PCA removed the collinearity sections among the seven SAE indices and extracted 92.47% of the information from these seven indices, while RA removed both the collinear section and random error. When the spectrum based on the PCA was used, the positive and negative REs of the plant species on the SAE were clearly identified. When there may be many random errors in the data, the spectrum based on the RA is recommended.

**Figure 5:**
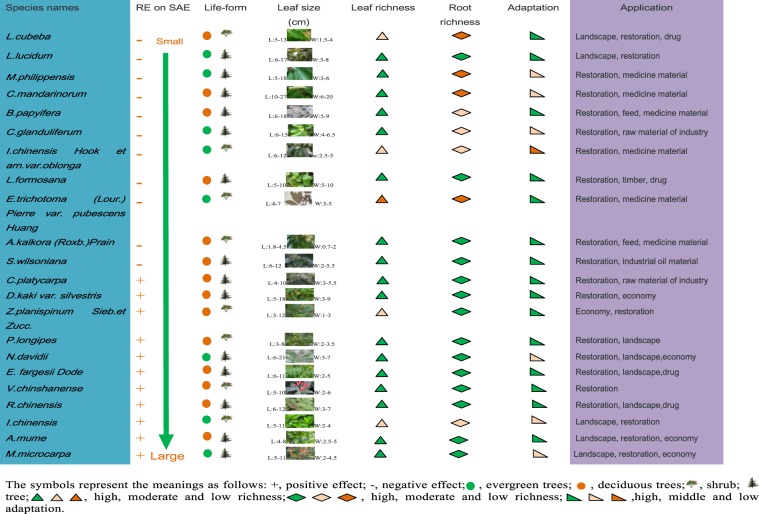
Botanical characteristics of eleven negative- and positive-effect species. The capital letters L and W represent the length and width of leaves, respectively. When the figure is enlarged to approximately 400%, the details of the leaves in the image can be seen.

The spectrum based on the PCA indicated that there were different species with negative and positive REs on SAE. After comparing the eleven species with the largest positive and negative effects, we found that the negative-effect species were primarily evergreen plant species, whereas the positive-effect species were mostly deciduous plant species (Fig. 5). The negative-effect species had relatively larger (length and width) and fewer leaves than did the positive-effect species^20^. All leaves of deciduous plants constitute litter and cover the soil after autumn every year, which is beneficial to the control of erosion. These positive-effect species also have relatively larger root systems than do negative-effect species. These characteristics effectively control aboveground and belowground soil erosion. These studied species are globally distributed, and related knowledge can be applied to karst regions worldwide^20^. However, the landscape, nutrient requirements and resistance to drought should also be comprehensively considered in the application of these results. For example, *C. platycarpa* is a leguminous species that often grows in barren landscapes because of its capability to fix nitrogen. Conversely, *P. longipes* requires a relatively fertile habitat for growth. Because *V. chinshanense* is a pioneer species, it is primarily adapted to dry habitats, while *N. davidii* thrives only in wet habitats for the improvement of community structure^20^. In general, plant species with different REs on SAE have respective ecological features and the capability to grow in specific habitats.

### BAM and AOAs and factors of interspecific variation in REs on SAE

Previous work has shown that root mucilage, polysaccharides, polygalacturonic acid and soluble carbohydrates in the rhizosphere strengthen SAE, but there is no information on the effects of the content or type of BAM and AOAs^22,38^. BAM acts as a carbon source of the small molecules preferentially used by microbes to synthesize other organic matter that may be used to produce hyphae for binding soil particles. BAM can also directly adhere soil particles^9^. Our results showed that the species that exerted positive effects on SAE in the spectrum commonly had high BAM contents in their rhizosphere; the negative-effect species exhibited the opposite result. Therefore, the content of BAM is one determinant of the REs on SAE, which validated our assumption. However, BAM accounted for only approximately 1/4 of the REs on SAE. Other factors, such as total organic matter and physical compression of root growth, also played a role in the REs on the SAE^12^. Specifically, the positive-effect species commonly presented high organic matter contents and good aggregation statuses in their rhizosphere and grew while depositing increased amounts of litter, increasing the organic matter content in the soil; these species also had vigorous root systems (Fig. 5). Consequently, the binding of soil particles with long molecules of organic matter and the compression of rhizosphere soil with roots strengthened, increasing the REs on SAE^34^. The exceptional species *M. microcarpa* and *A. mume* directly support this point. *M. microcarpa* and *A. mume* had high contents of organic matter and vigorous root systems in their rhizospheres. Moreover, the comprehensive SAE index was largest for these species, but the BAM contents were not high (Fig. 1). These results indicated that factors other than BAM had REs on the SAE^9^.

By comparison, AOAs had a non-significant negative effect on the REs on SAE. AOAs can limit microorganism growth, which weakens the binding of soil particles, leading to a decrease in the REs on SAE^39–41^. AOAs are also soil dispersants that can break down microaggregates, decreasing the REs on SAE^42^. Consequently, AOAs might have played a negative role in SAE. In addition, the negative-effect species along the spectrum of the comprehensive index of the REs on SAE presented high contents of AOAs and low contents of both BAM and total organic matter in their rhizospheres. The leaves and root systems of these species were also not especially dense or vigorous (Fig. 5). Together, these results support the inference of the negative REs of these plant species on SAE.

## Conclusions

The globally distributed and key woody species investigated in this study have different REs on SAE in the Guizhou karst region of China. Most plant species exert comprehensive positive REs on SAE. PCA and RA were used to combine the respective SAE indices into a comprehensive index to identify the REs of each species on the SAE. We established a spectrum to describe the sizes of the REs on the SAE for the different plant species. Along the spectrum, eleven species with negative REs on SAE and eleven species with high REs on SAE were directly identified for applications to control underground soil leakage. The spectrum directly indicated the interspecific variation in the REs on the SAE. The BAM in the rhizosphere accounted for 16–23% of the variation in REs on SAE, but AOAs exerted negative REs on the SAE. Plant species with high REs on SAE were found to contain high concentrations of BAM in their rhizospheres, and most of these plant species are deciduous, with dense leaves and vigorous root systems. Those species with positive REs on SAE and other excellent botanical characteristics are worthy of continued investigation.

## Materials and Methods

### Study area and key species

**Figure 6:**
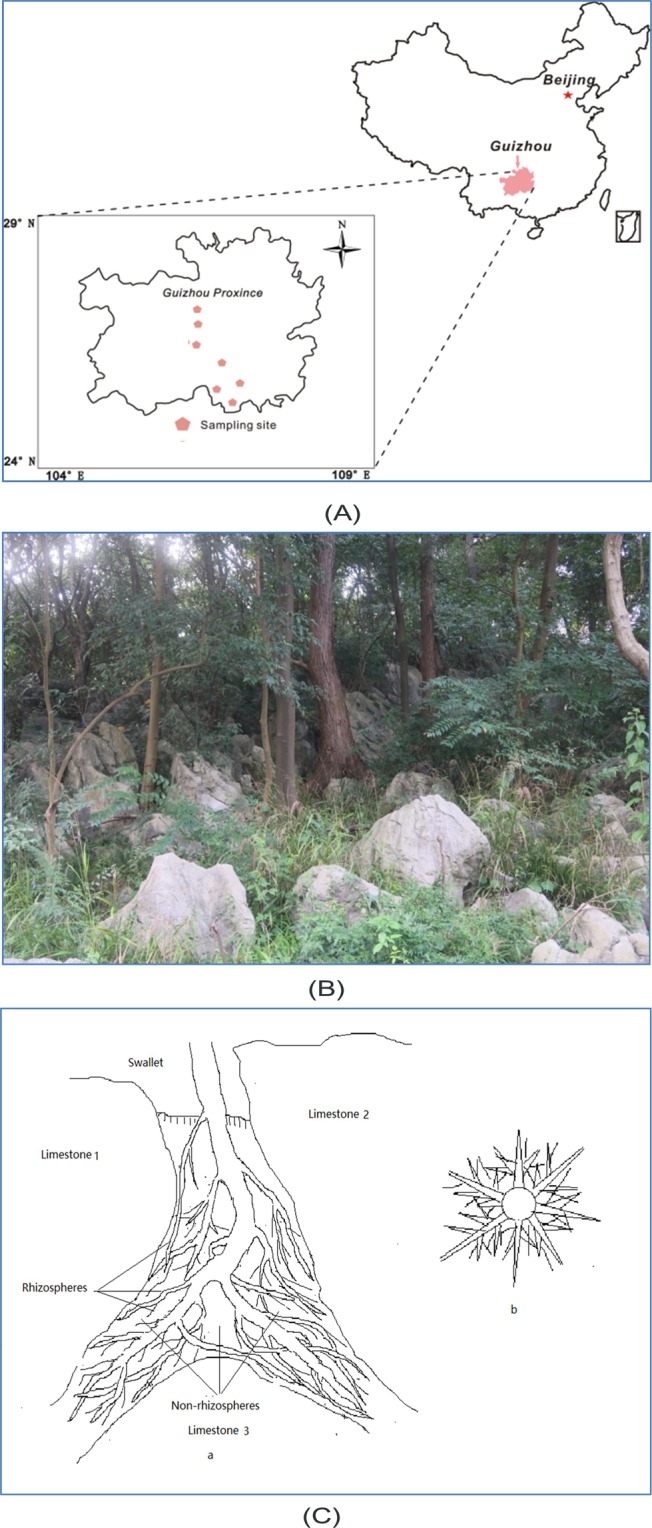
Sampling sites. (**A**) Locations of sampling sites; (**B**) plants growing in fissures; (**C**) the root system of a plant growing in a fissure. In (C), a and b represent the longitudinal and cross-sectional sections of root systems, respectively.

This study was performed in the area surrounding Guiyang city, Guizhou Province, China (26º17′-26º22′ N, 106°07′-107°17′ E; 506–1762 m), which is characterized by karst formations and seminatural forests. We selected 42 plant species with key roles in structuring the karst evergreen broadleaf forests and seven forest sites (Fig. 6) where these species occur, in accordance with Huang *et al*.^20^. These species belong to 21 families (Supplementary Material S3) and include both evergreen (22) and deciduous (20) species. The species are dominant or edificator species and are usually rooted within fissures on the karst substrate (Fig. 6B, Supplementary Material S4). The root systems of these species filled nearly all the spaces in the soil fissures (Fig. 6C), thereby playing a major role in all soil processes.

### Soil sampling

We identified seven forest sites (Fig. 6) in which the target species were known to occur according to previous studies^20^. Afterward, 20 m × 20 m plots with similar habitats were established in each site. We successively searched for three individual plants of each species in each plot according to the typical conditions of the adult plants, ensuring similar ages (15–20 years old; at such an age, plants vigorously grow with a high secretion of root exudates), canopy coverage, tree height, and diameter at breast height. If three similar individuals of one species were not located within a plot, we continually searched in subsequent plots. The final number of plots within the 7 forest sites was 129. After the identification of three individuals per species, the soil in the fissure was removed in successive layers. After finding living fibrous roots, we used the stripping method to remove the external (thickness > 1 cm) soil around the roots and collected 500 g of inner soil (0–1 cm thickness) per tree^13^. The sampling diameter of the living roots was less than 0.7 cm, so the roots could vigorously and continually release exudates. A 500 g soil sample from the non-rhizosphere zones was also collected. After they air dried for one week, the soil samples were sieved through 2 mm and 0.25 mm sieves. All the field experiments were approved by the Administration Bureau of Two Lakes and One Reservoir in Guiyang.

### Tests of SAE and the REs on the SAE

We selected seven SAE indices that are currently widely applied to SAE research; information concerning these SAEs is shown in Table 2^35,36^. The aggregate status and degree refer to the aggregating capability of soil particles with a diameter > 50 μm, which were quantified to ensure that the empirical values and percentages reflected good soil structure. The dispersion ratio and dispersion coefficient refer to the dispersion characteristics of soil microaggregation at diameters of <50 μm and 2 μm. A high ratio and coefficient for the two indices are not beneficial for improving soil structure. The particle fractal dimension and microaggregation fractal dimension indicate whether the physical nature of the soil is beneficial to soil stability. The soil organic matter content indicates how much matter can be used to adhere to soil particles to improve soil stability. All seven indices represent different physical natures of the soil in terms of stability. For each soil sample, we quantified the seven SAE indices via formulas 1–5 after testing the water-stable aggregates by the wet-screening method and the microaggregates and mechanical components by the pipette method^35,36^. The wet-screening method was performed as follows: soil samples were first placed on the uppermost layer of an electrokinetic wet-screening machine that contained several layers of sieves; water was used then to continually dissolve the soil sample in the uppermost layer; the electrokinetic wet-screening machine continued to stir and cause the soil particles to pass through the sieves with different aperture sizes; last, the soil particles in each sieve were collected, dried and weighed to determine the percentages of the water-stable soil particles with different diameters. The microaggregates were directly tested after the soil samples had been dissolved and washed through a 0.25 mm sieve. Since the mechanical components are the basic components of soil particles, the soil samples needed to be dissolved in distilled water and treated with a soil dispersing agent, sodium hexametaphosphate solutions before being tested^43^. The organic matter content (x_5_) was tested with the potassium dichromate volumetric method in accordance with the national standards of China. The method involves the oxidization of soil samples with standardized potassium dichromate-sulfuric acid solution; excessive potassium dichromate-sulfuric acid solution was neutralized by titration of standardized ferric sulfate, and the organic matter content was calculated on the basis of the consumed potassium dichromate-sulfuric acid solution^35,36^.

1 $$\begin{array}{ccc}{\rm{Aggregate}}\,{\rm{status}}\,({{\rm{x}}}_{1}, \% ) & = & ({\rm{microaggregates}}\,{\rm{with}}\,{\rm{a}}\,{\rm{diameter}}\, > 50\,{\rm{\mu }}{\rm{m}}, \% )\\  &  & -\,({\rm{soil}}\,{\rm{mechanical}}\,{\rm{components}}\,{\rm{with}}\,{\rm{a}}\,{\rm{diameter}}\,\\  &  &  > 50\,{\rm{\mu }}{\rm{m}}, \% )\end{array}$$

2 $${\rm{Degree}}\,{\rm{of}}\,{\rm{aggregation}}\,({{\rm{x}}}_{2}, \% )=\frac{Aggregate\,status\times 100}{Micro \mbox{-} aggregates\,with\,a\,diameter > 50\,\mu m}$$

3 $${\rm{Dispersion}}\,{\rm{ratio}}\,({{\rm{x}}}_{3}, \% )=\frac{Micro \mbox{-} aggreates\,with\,a\,diameter < 50\,\mu m\times 100}{Soil\,mechanical\,components\,with\,a\,diameter < 50\,\mu m}$$

4 $${\rm{Dispersion}}\,{\rm{coefficient}}\,({{\rm{x}}}_{4}, \% )=\frac{{\rm{Micro}} \mbox{-} {\rm{aggreates}}\,{\rm{with}}\,{\rm{a}}\,{\rm{diameter}} < 2\,{\rm{\mu }}{\rm{m}}\times 100}{{\rm{Soil}}\,{\rm{mechanical}}\,{\rm{components}}\,{\rm{with}}\,{\rm{a}}\,{\rm{diameter}} < 2\,{\rm{\mu }}{\rm{m}}}$$

5 $$3 \mbox{-} {\rm{D}}=\,{\rm{lg}}\,(\frac{W(\delta  < {\bar{d}}_{i})}{{W}_{0}})/{\rm{lg}}\,(\frac{{\bar{d}}_{i}}{{d}_{{\rm{\max }}}})$$

Here, *3-D* is the fractal dimension (x_6_ or x_7_);$\,{\rm{W}}({\rm{\delta }} < {\bar{d}}_{i})$ is the cumulative weight of all the sieve grades of soil particles less than ${\bar{d}}_{i}$ in diameter; *δ* is the soil particle diameter; ${\bar{d}}_{i}$ is equal to$\,({d}_{i}+{d}_{i+1})/2$; ${d}_{i}$ and *d*_i+1_ are the average diameters of the *i* and *i* + 1 sieve grades of soil particles, respectively; d_max_ is the average diameter of all the soil particles whose diameters are greater than the maximum sieve grade; and *W*_0_ is the total weight of all the sieve grades of soil particles. Thereafter, the REs of each species on SAE were quantified via formula 6.

6 $$RE={V}_{{\rm{r}}}-{V}_{{\rm{n}}}$$

where *V*_r_ and *V*_n_ represent the values of the SAE indices in rhizosphere and non-rhizosphere zones, respectively.

The CV was used to test the differences in each of the SAE indices among the 42 plant species because F- and t-tests could not be applied. The CV (%) is equal to s × 100/µ. When CV > 30%, there was a statistically significant difference among plant species; when CV < 30%, the significance level was determined by the CV_u_, which is the upper confidence limit of the CV. When CV < CV_u_, no significant statistical variation between plant species was detected. Here, CV_u_ is equal to $\{{{\rm{CV}}}_{{\rm{u}}}=\{{{\rm{CV}}}_{1-{\rm{\alpha }}}^{2}({\rm{n}}-1)[1+\frac{{{\rm{CV}}}^{2}({\rm{n}}-1)}{{\rm{n}}}]\}/[(n-1){{\rm{CV}}}^{2}]$, where ${{\rm{CV}}}_{1-{\rm{\alpha }}}^{2}({\rm{n}}-1)$ was obtained by searching the quantiles of the chi-squared distribution where the degrees of freedom is equal n − 1 and where the probability is equal to 1 − α^44^.

### BAM measurements

We measured the BAM in the rhizosphere soil samples, including the total sugars, total amino acids, phenolic compounds, and free amino acids, using anthracenone colorimetry, tri-ketone colorimetry and Folin-Ciocalteu colorimetry^32^.

### GC-MS testing of AOAs

We sieved 40 g of the rhizosphere and non-rhizosphere soils of each species through a 40-mesh sieve. The root exudates in these soils were extracted by dichloromethane and ultrasonic waves at 1200 watts^45–47^. The suspension solution after extraction was then filtered, concentrated, dissolved by ether, after which it ultimately became the solution to be tested by GC-MS^45–47^ (see the specific steps in Supplementary Material S5). The test results of soil samples collected from the non-rhizosphere of a plant species for each type of AOA were then deducted from the results of soil samples collected from the rhizosphere.

### Spectrum of the comprehensive REs on SAE

The REs on SAE were investigated using the above seven SAE indices, which provided respective measures and basic information. However, if all seven indices were used to assess the REs of each species on the SAE, as shown in Table 2, the information would have been fragmented. Conversely, using only one index among all seven SAE indices was not optimal. Therefore, the combination of the information from the seven SAE indices for each species into an optimal normalized index was necessary to compare the interspecies variation in the REs on the SAE. To this end, we utilized PCA and RA to combine these indices into a comprehensive index in which the collinear information from the seven SAE indices was eliminated by mathematical processes^48,49^. Two spectra describing the REs of each species on SAE were subsequently established by sequencing the magnitude of the comprehensive index.

Various spectra, such as the light spectrum and life form spectrum, have been widely used in physics, chemistry and biology to describe changes in processes^50^. The spectrum of the REs on SAE is a new tool for quantifying the relative contribution of plant species to the stability of soils in their rhizosphere. Thus, the spectrum can provide a basis for selecting plant species to be used in ecological engineering for controlling soil erosion by underground leakage. The specific mathematical methods used were as follows:

#### PCA

We assumed that the *k* (*k* ≤ *p*) comprehensive indices *Y*_1_,…,*Y*_*k*_ represented *p* observable SAE indices *X* (formula 7).

7 $${Y}_{i}={L}_{i}\times X$$

where *L*_*i*_ is the characteristic vector corresponding to the eigenvalue of the covariance matrix of *X* (*i* = 1, …, *k*). For formula 7, the standard deviation (*SD*) (*Y*_*i*_) is as large as possible, but *Cov* (*Y*_i_, *Y*_*j*_) = 0, *i* ≠ *j*^48,49^. After expansion, formula 7 changes to:

8 $$\begin{array}{c}{{\rm{Y}}}_{1}={{\rm{L}}}_{11}{{\rm{x}}}_{1}+{{\rm{L}}}_{21}{{\rm{x}}}_{2}+\cdots +{{\rm{L}}}_{{\rm{p1}}}{{\rm{x}}}_{{\rm{p}}}\\ {{\rm{Y}}}_{2}={{\rm{L}}}_{12}{{\rm{x}}}_{1}+{{\rm{L}}}_{22}{{\rm{x}}}_{2}+\cdots +{{\rm{L}}}_{{\rm{p2}}}{{\rm{x}}}_{{\rm{p}}}\\ \cdots \\ {{\rm{Y}}}_{{\rm{k}}}={{\rm{L}}}_{{\rm{1k}}}{{\rm{x}}}_{1}+{{\rm{L}}}_{{\rm{2k}}}{{\rm{x}}}_{2}+\cdots +{{\rm{L}}}_{{\rm{pk}}}{{\rm{x}}}_{{\rm{P}}}\end{array}$$

where *x*_1_, *x*_2_, …, *x*_*p*_ are the values of the SAE indices in *X*^48,49^.

We used formula 9 to calculate *Y*, which represented the comprehensive REs of each species. *λ*_*i*_. presents the variance contribution of *Y*_*i*_, and *i* = 4.

9 $${\rm{Y}}=\mathop{\sum }\limits_{{\rm{i}}=1}^{{\rm{k}}}\,\frac{{\rm{\lambda }}{\rm{i}}}{{\sum }_{{\rm{i}}=1}^{{\rm{k}}}{{\rm{\lambda }}}_{{\rm{i}}}}{{\rm{Y}}}_{{\rm{i}}}$$

Last, the spectrum of the REs of key woody species was presented as a histogram of gradually increasing values of *Y* calculated for each species (the calculation processes are available in Supplementary Material S6).

#### RA

We used *Y* to represent the comprehensive REs of each species on the SAE and *X* for the seven indices of SAE^48,49^. *Y* and *X* are random vectors for different species. Moreover, *E*(*Y*) and *E*(*X*) are close to zero. Thus, there is a linear operator *Q*_k_ that can make *X* change into *Q*_k_*X*^48,49^. *Y* may be further defined by regression formula 10 on *Q*_k_*X*.

10 $${\rm{Y}}={\rm{R}}({{\rm{Q}}}_{{\rm{k}}}{\rm{{\rm X}}})+{\rm{\varepsilon }}$$

where *R* is the regression coefficient and where ɛ is the random error.

We can further write the linear section of formula 10 as follows:

11 $$\widehat{Y}=R({Q}_{k}X)$$

When the variance in *Y* is explained maximally by $\widehat{Y}$, $\widehat{Y}$ can be used to describe the comprehensive REs of each species^48,49^.

The spectrum of the REs was presented by a histogram of the gradually increasing values of $\widehat{Y}$ calculated for each species. The degree to which $\widehat{Y}$ explains *Y* is defined as the redundancy index (Supplementary Material S7).

## Supplementary information

Supplenmentary Material.

## Data Availability

The datasets generated and/or analyzed during the current study are available from the corresponding author on reasonable request.
