# Supplementary material for: An interspecific variation in rhizosphere effects on soil anti-erodibility
Source: Sci Rep. 2020 Feb 12;10:2411. doi: 10.1038/s41598-020-58784-z (PMC7015890; doi:10.1038/s41598-020-58784-z)
Supplement: Supplementary file 1 — Supplenmentary Material. [file 41598_2020_58784_MOESM1_ESM.pdf]

An interspecific variation in rhizosphere effects on soil anti-erodibility

Zhenhong Wang[1*], Alessandro Chiarucci[2], Hong Fang[3], Mouhui Chen[1]

[1]Key Laboratory of Subsurface Hydrology and Ecological Effects in Arid Regions of the Ministry of Education; School of Environmental Science and Engineering, Chang'an University, Xi'an, China

[2]Department of Biological, Geological and Environmental Science, University of Bologna, Via Irnerio 42-40126, Bologna, Italy

[3]Water-affair Authority of Xifeng County, 551100, Guiyang, China

*Corresponding author: Zhenhong Wang, E-mail: w_zhenhong@126.com

Contents

S1: Distribution of karst landscape worldwide……………………………………………………………………………..3

S2: Statistical summaries of the contents of BAM and AOA………………………………………………………………... 13

S3: Key woody plant species in karst forests………………………..14

S4: Photos of study sites………………………………………………………………………………………………….16

S5: Specific steps of GC-MS analysis for root exudates…………………………………………………………………21

S6: Results and processes of principal component analysis……………………………………………………………...22

S7: Results and processes of redundancy analysis……………………………………………………………………….27

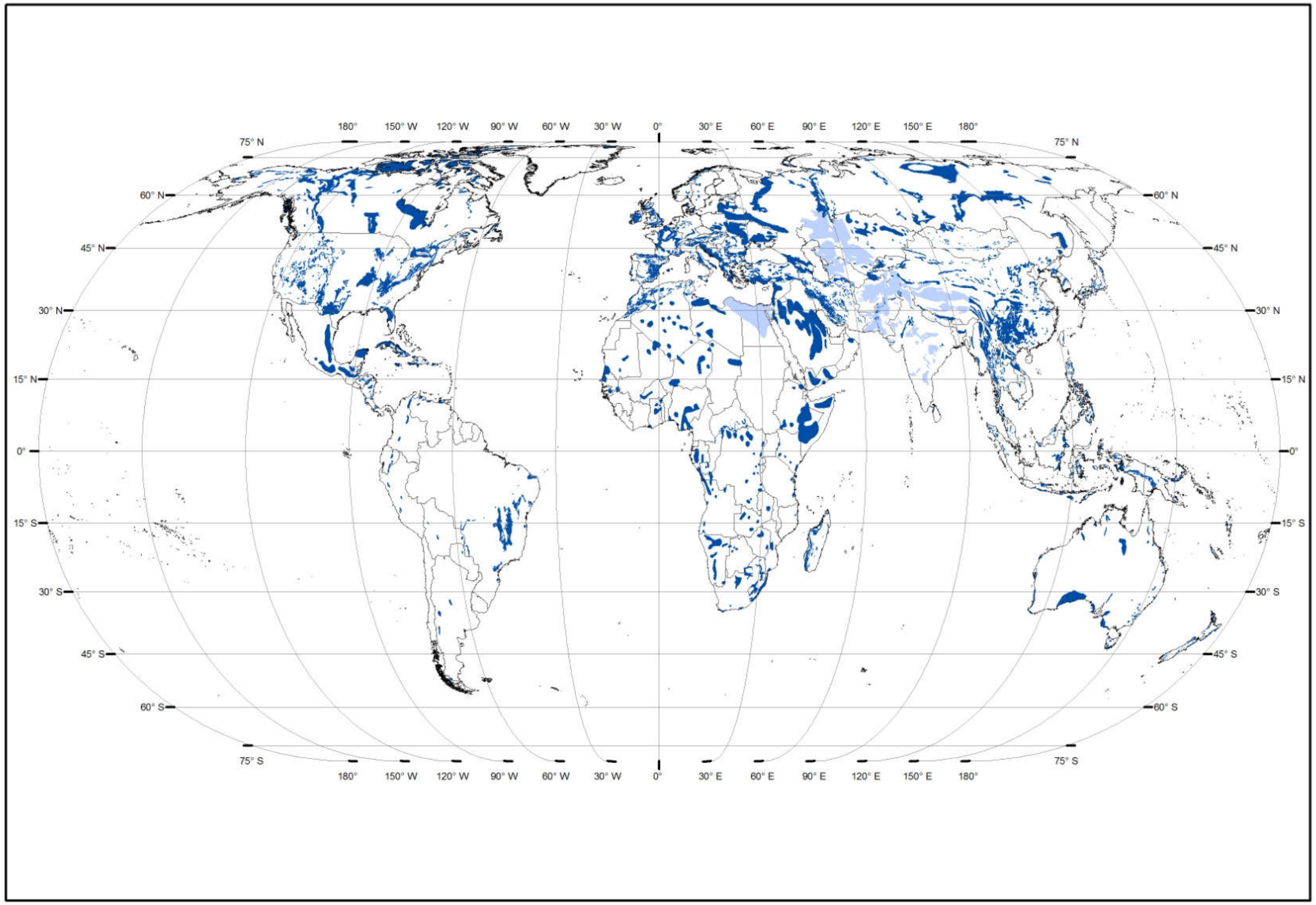

S1 A: Distribution of karst landscape worldwide. Blue region represents karst regions. The same below

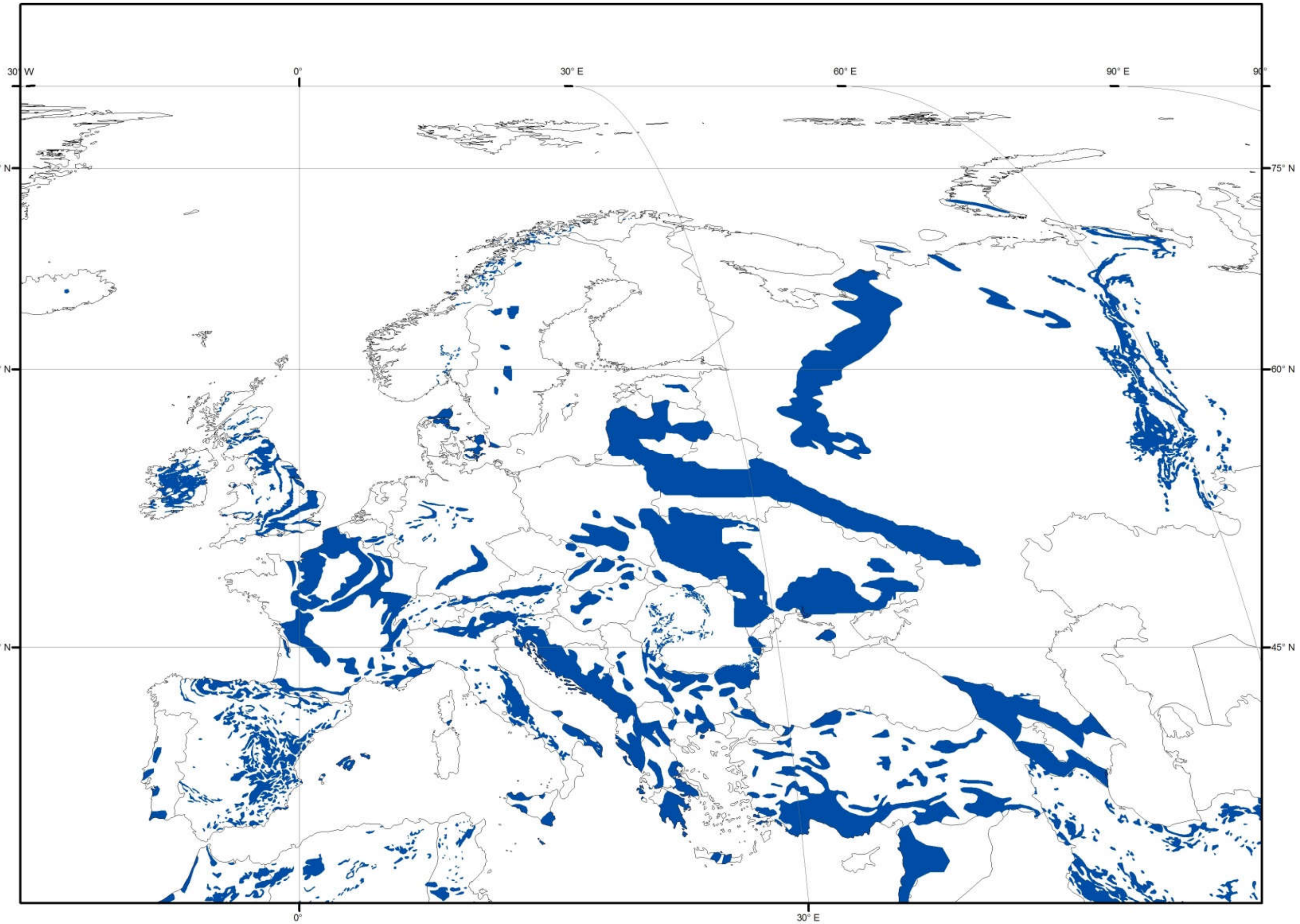

S 1 B: Distribution of karst landscape in Europe

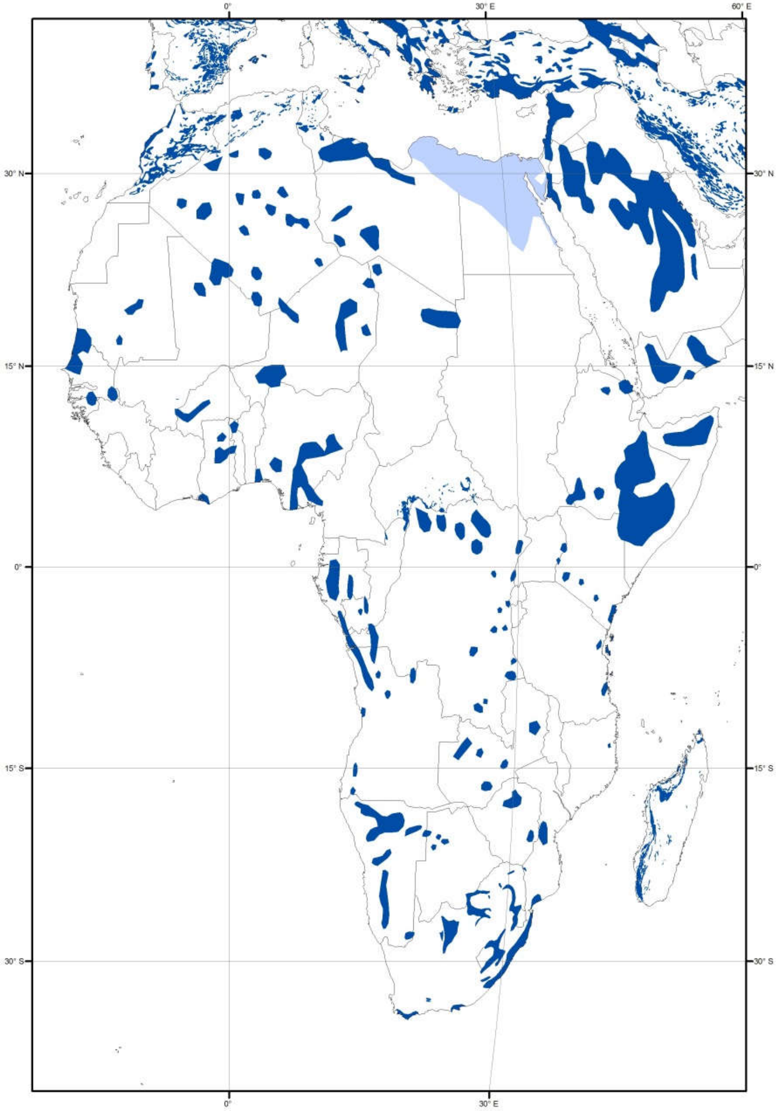

S 1 C: Distribution of karst landscape in Africa

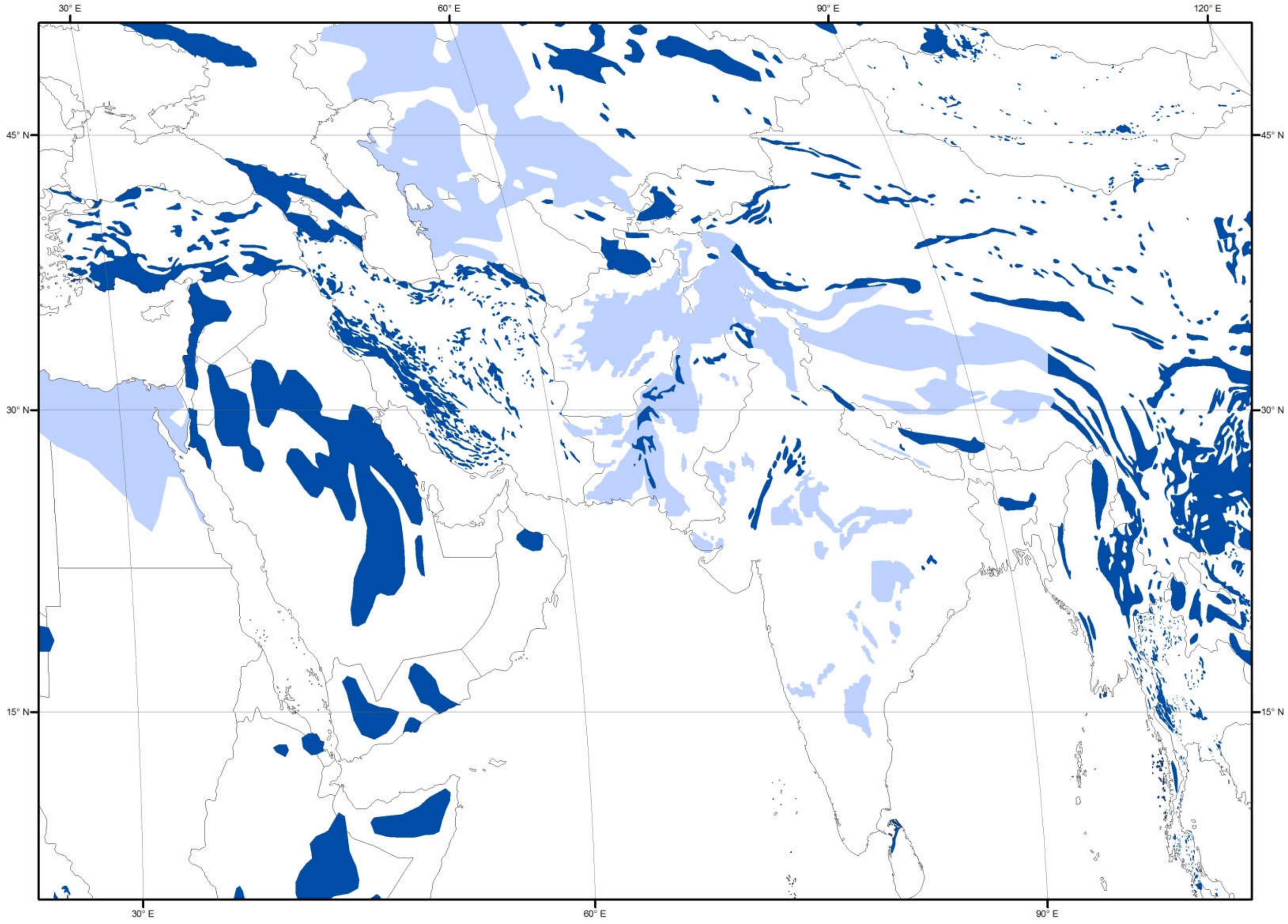

S 1 D: Distribution of karst landscape in central Asia and India

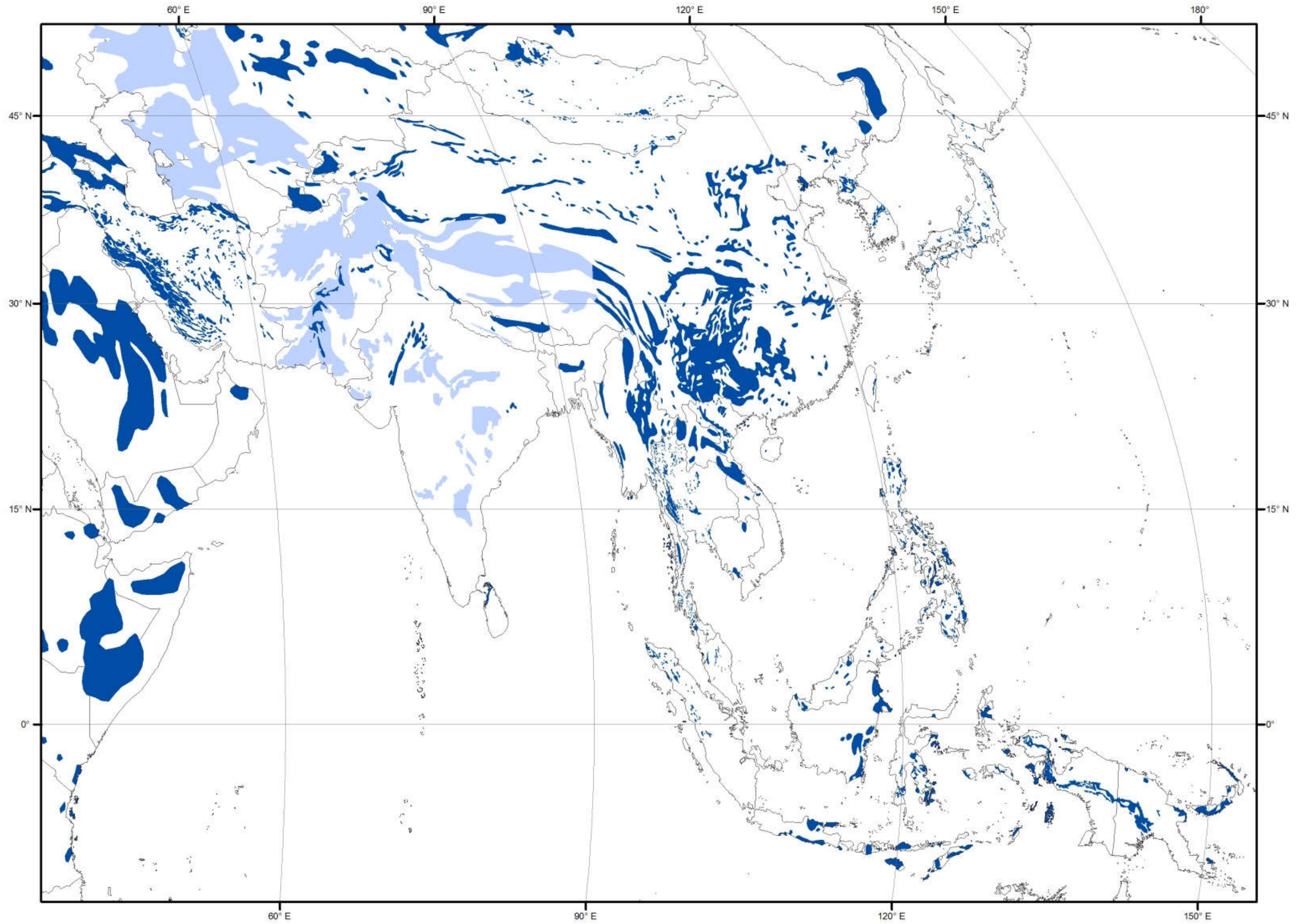

S 1 E: Distribution of karst landscape in east and southeast Asia

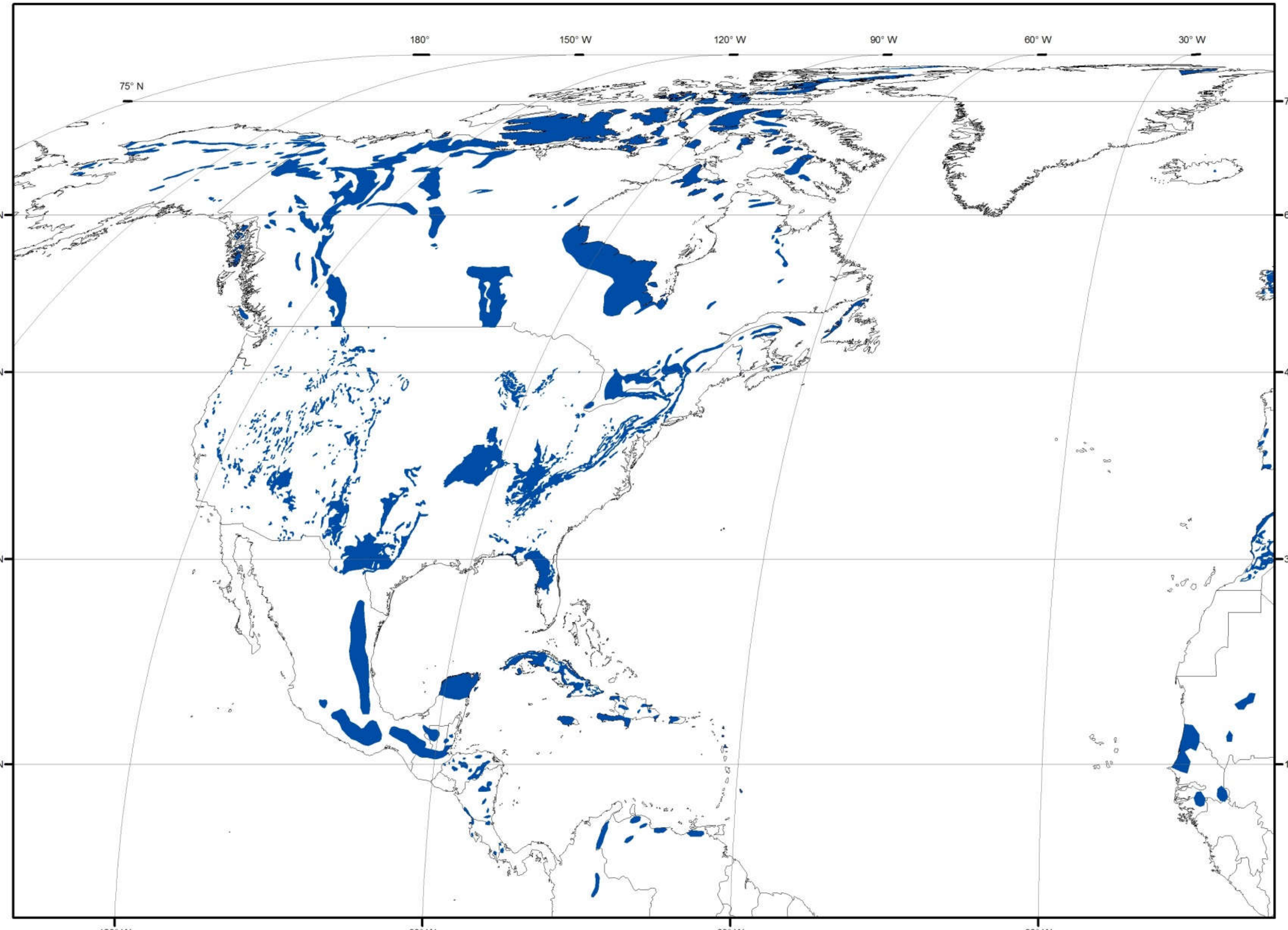

S 1 F: Distribution of karst landscape in north America

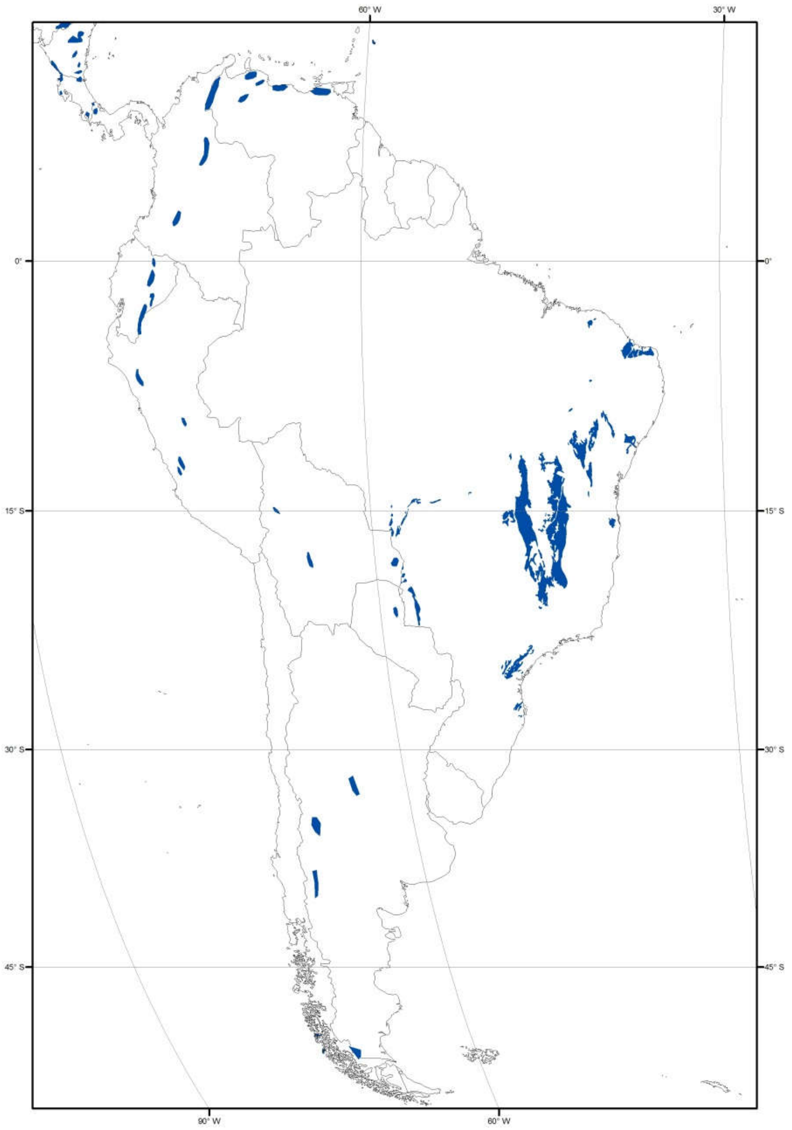

S 1 G: Distribution of karst landscape in south America

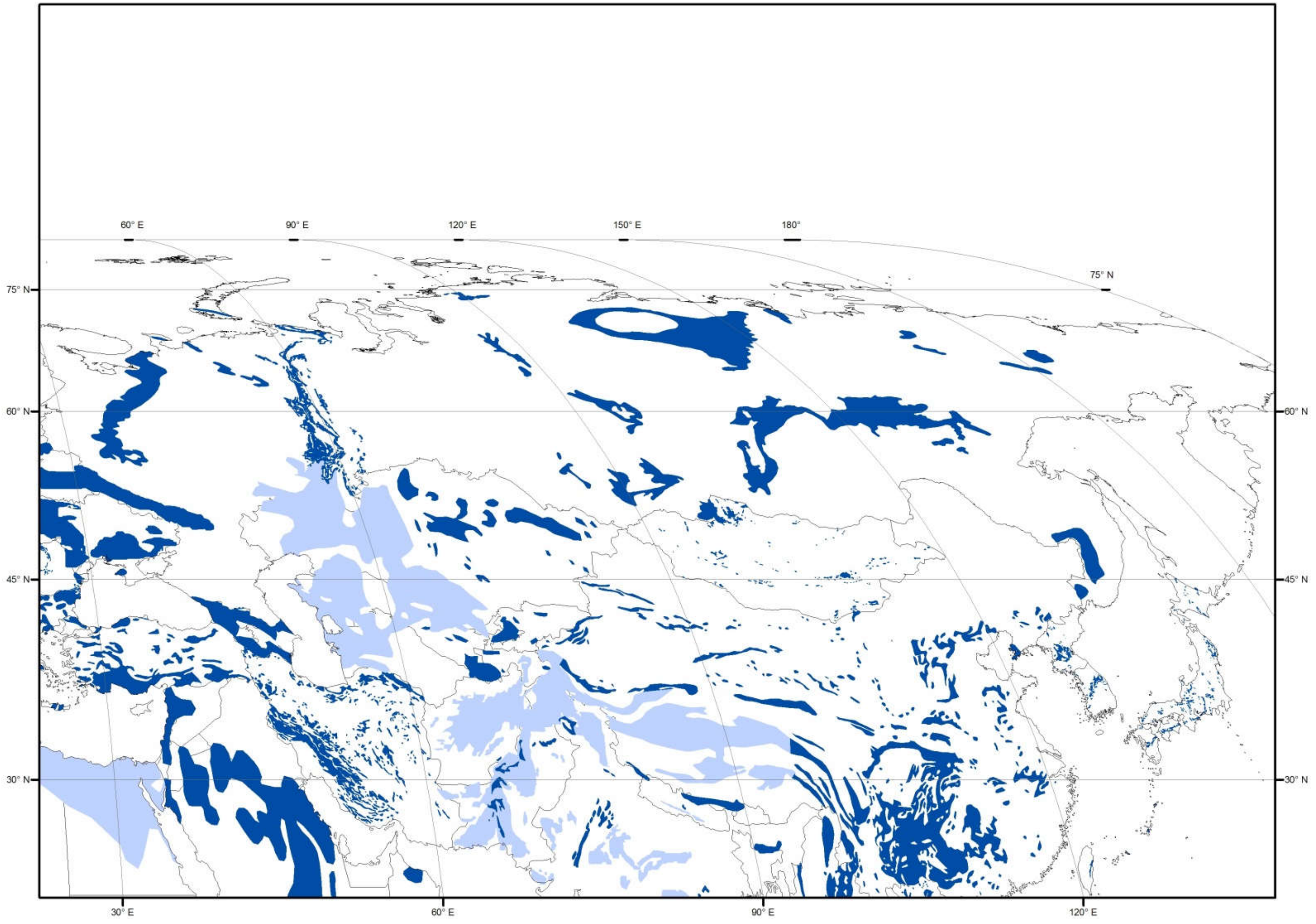

S 1 H: Distribution of karst landscape in Russia

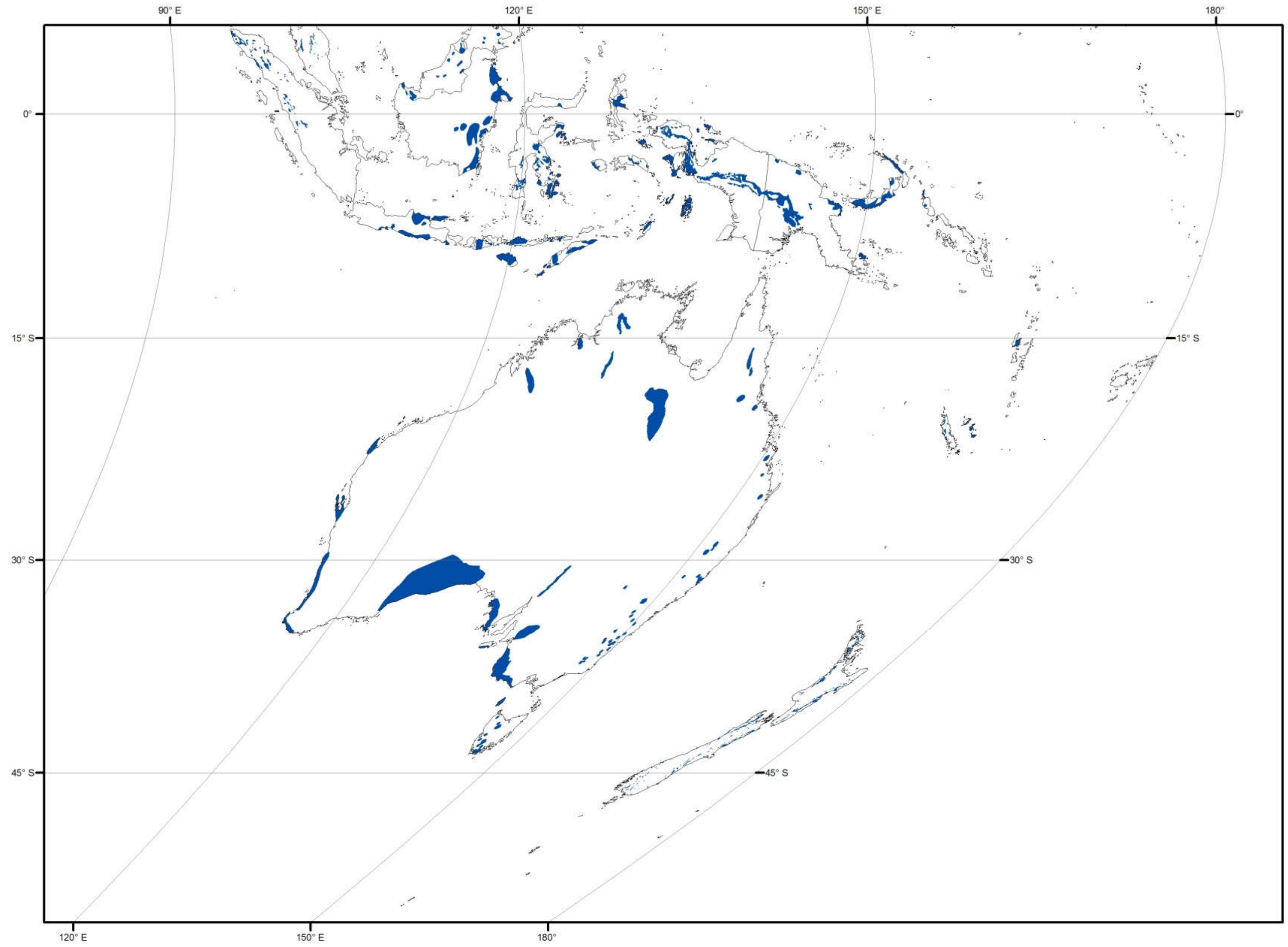

S 1 I: Distribution of karst landscape in Austria and south Asia

Data cited from Karst Scientific Data Center:

http://www.karstdata.cn/

S2: Statistical summaries of the contents of BAM and AOA

BAM	Total dissolved sugar	Total amino acids	Phenolic compound	Free amino acid
Maximum (g/kg)	2.26	1.04	3.81	0.01835
Minimum (g/kg)	0.27	0.17	0.81	0.00286
Mean (μ, g/kg)	1.06	0.56	2.52	0.01010
Variance (σ)	0.23	0.09	0.82	0.00002
Standard deviation (s)	0.48	0.29	0.90	0.00397
Coefficients of variation (CV)	45.75	52.32	35.83	39.33
n	42	42	42	42

AOA	Amides	Phenolic ether	Aldehyde	Ketone	Total
Maximum (%)	11.91	20.57	2.61	11.70	92.01
Minimum (%)	0.44	1.12	0.23	1.04	67.28
Mean (μ, %)	4.36	6.35	1.25	4.23	82.11
Variance (σ)	10.54	40.71	0.41	7.62	50.45
Standard deviation (s)	3.25	6.38	0.64	2.76	7.10
Coefficients of variation (CV)	74.41	100.47	50.92	65.31	8.65
n	19	19	19	19	19

n: the number of plant species. Total number of tested plants was 42 but it was possible to detect AOA only from the rhizospheres of 19 species (for the other 23 species the values were below the instrumental detection limits)

S3: Key woody plant species in karst forests

Tree species	Family	Genus	Lifeform
Ligustrum lucidum	Oleaceae	Ligustrum L	Evergreen tree
Ilex chinensis	Aquifoliaceae	Ilex	Evergreen tree
Cinnamomum glanduliferum	Lauraceae	Cinnamomum	Evergreen tree
Machilus microcarpa	Lauraceae	Machilus	Evergreen tree
Lithocarpus glaber (Thunb.) Nakai	Fagaceae	Lithocarpus	Evergreen tree
Cyclobalanopsis glauca	Fagaceae	cyclobalanopsis	Evergreen tree
Lithocarpus confinis Huang	Fagaceae	Lithocarpus	Evergreen tree or dungarunga
Diospyros cathayensis	Ebenaceae	Diospyros L	Evergreen dungarunga
Cyclobalanopsis gracilis	Fagaceae	cyclobalanopsis	Evergreen dungarunga
Itea yunnanensis	Saxifragaceae	escallonia	Evergreen shrub or dungarunga
Ilex corallina	Aquifoliaceae	Ilex	Evergreen shrub or tree
Lindera communis	Lauraceae	Lindera	Evergreen shrub or dungarunga
Mallotus philippensis	Euphorbiaceae	Mallotus	Evergreen shrub or dungarunga
Evodia fargesii Dode	Saxifragaceae	Evodia	Evergreen shrub or dungarunga
Ilex memecylifolia	Aquifoliaceae	Ilex	Evergreen shrub or tree
Elaeagnus pungens	Elaeagnaceae	Elaeagnus	Evergreen shrub
Zanthoxylum planispinum Sieb.et Zucc.	Rutaceae	Zanthoxylum L	Evergreen shrub
Pyracantha atalantioides	Rosaceae	Pyracantha	Evergreen shrub
Nothopanax davidii	Araliaceae	metapanax	Evergreen shrub
Clerodendrum mandarinorum	Verbenaceae	Clerodendrum	*Semi-evergreen shrub or tree*
Toddalia asiatica	Rutaceae	Toddalia	Evergreen woody vines
Quercus aliena	Fagaceae	Quercus L	Deciduous tree

Vitex canescens	Verbenaceae	Vitex L	Deciduous tree
Carpinus pubescens	Betulaceae	Carpinus	Deciduous tree
Platycarya longipes	Juglandaceae	Platycarya	Deciduous small tree
Cladrastis platycarpa	Leguminosae	Cladrastis	Deciduous tree
Albizia kalkora (Roxb.)Prain	Leguminosae	Albizzia	Deciduous tree
Celtis sinensis	Ulmaceae	Hackberry L	Deciduous tree
Broussonetia papyifera	Moraceae	Broussonetia	Deciduous tree
Diospyros kaki var. silvestris	Ebenaceae	Diospyros L	Deciduous tree
Catalpa fargesii f. duclouxii	Bignoniaceae	Catalpa L	Deciduous tree
Liquidambar formosana	Hamamelidaceae	liquidambar L	Deciduous tree
Swida wilsoniana	Cornaceae	Cornus	Deciduous tree
Armeniaca mume	Rosaceae	Armeniaca K.Koch	Deciduous small tree or shrub
Cudrania tricuspidata	Moraceae	Cudrania	Deciduous shrub or small tree
Rhamnella martinii	Rhamnaceae	Rhamnella	Deciduous shrub or small tree
Rhus chinensis	Anacardiaceae	Rhus L	Deciduous shrub or small tree
Litsea cubeba	Lauraceae	Liasea Lam	Deciduous shrub or small tree
Viburnum chinshanense	Caprifoliaceae	Viburnum L	Deciduous shrub
Ligustrum sinense	Oleaceae	Ligustrum L	Deciduous shrub or small tree
Evodia trichotoma (Lour.) Pierre var. pubescens Huang	Rutaceae	Evodia Forst	Deciduous shrub or small tree

S4: Photos of study sites

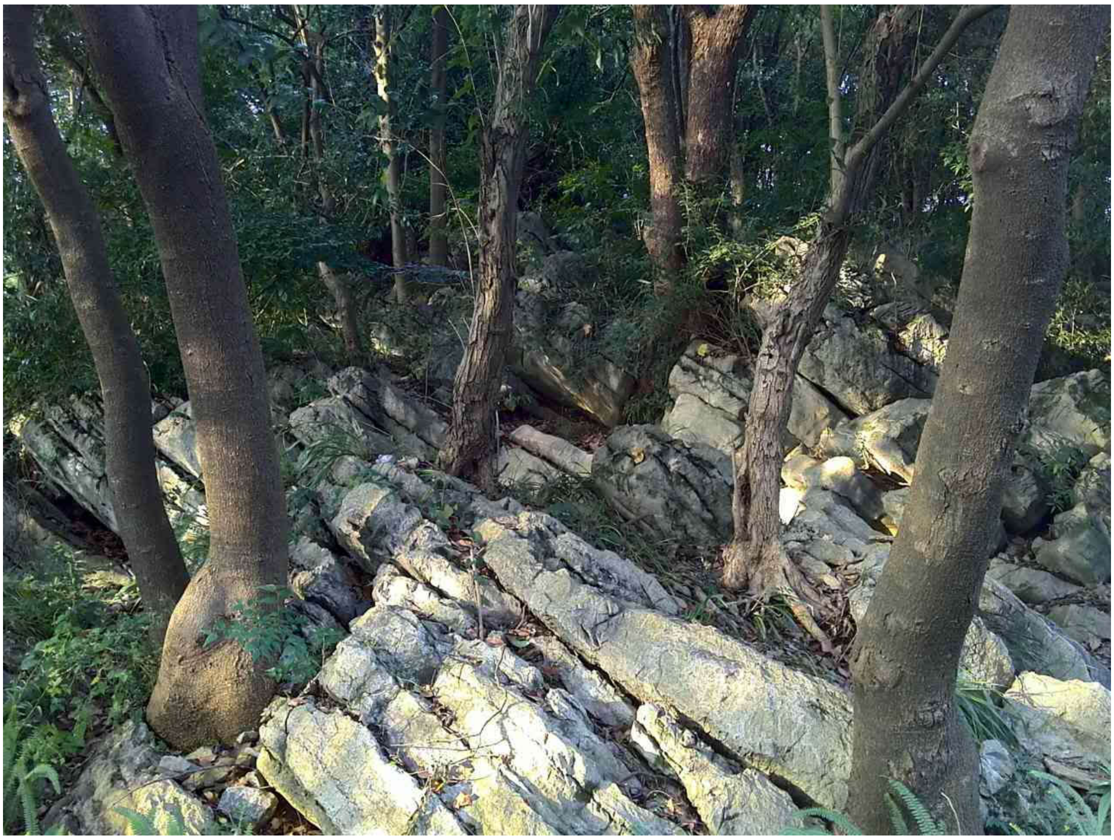

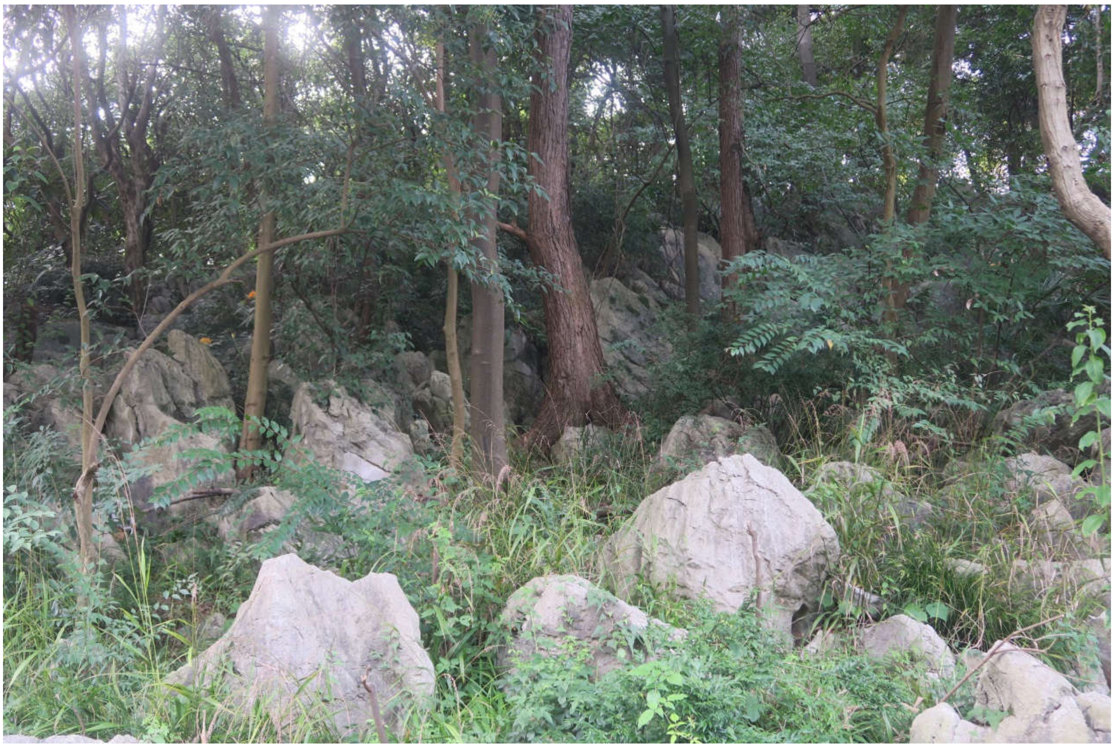

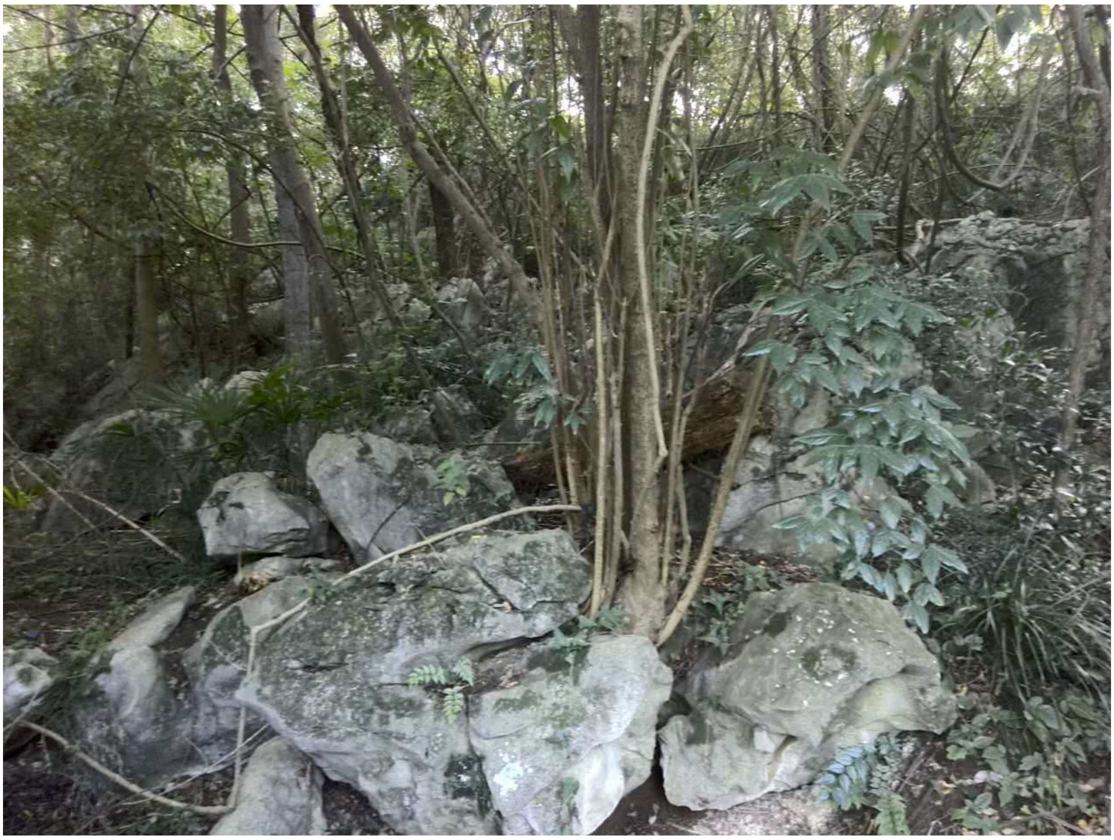

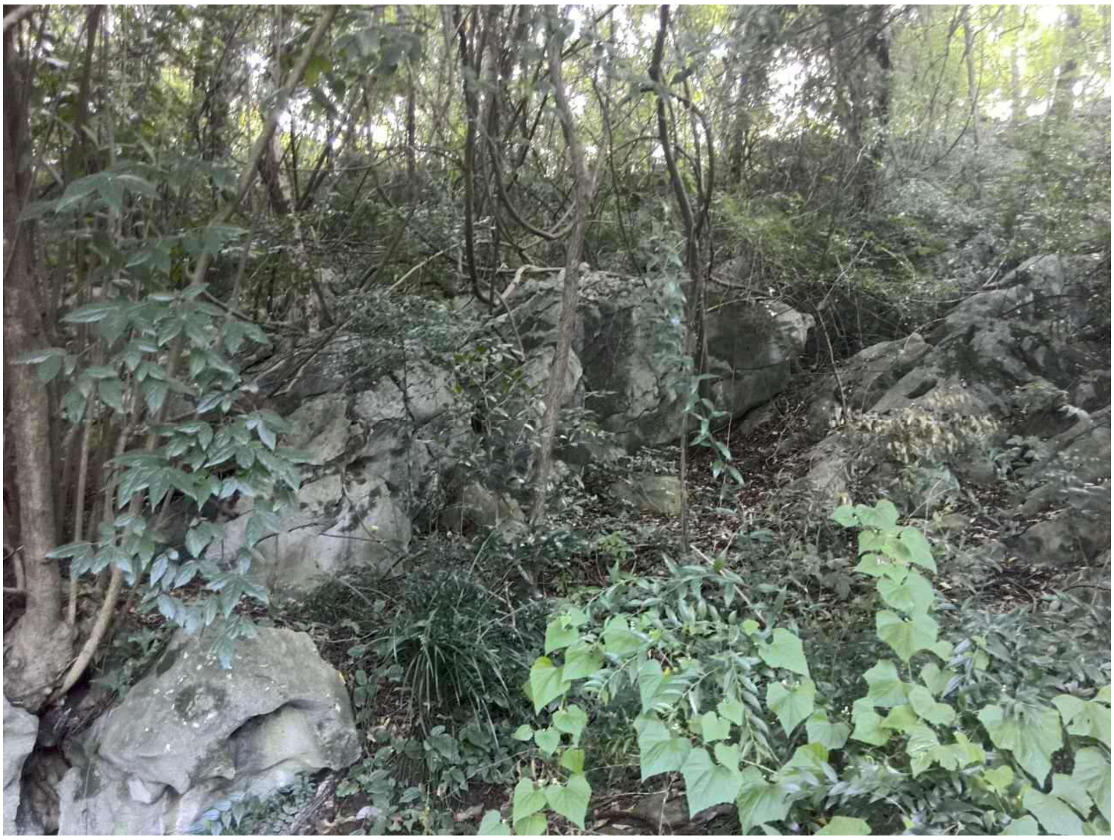

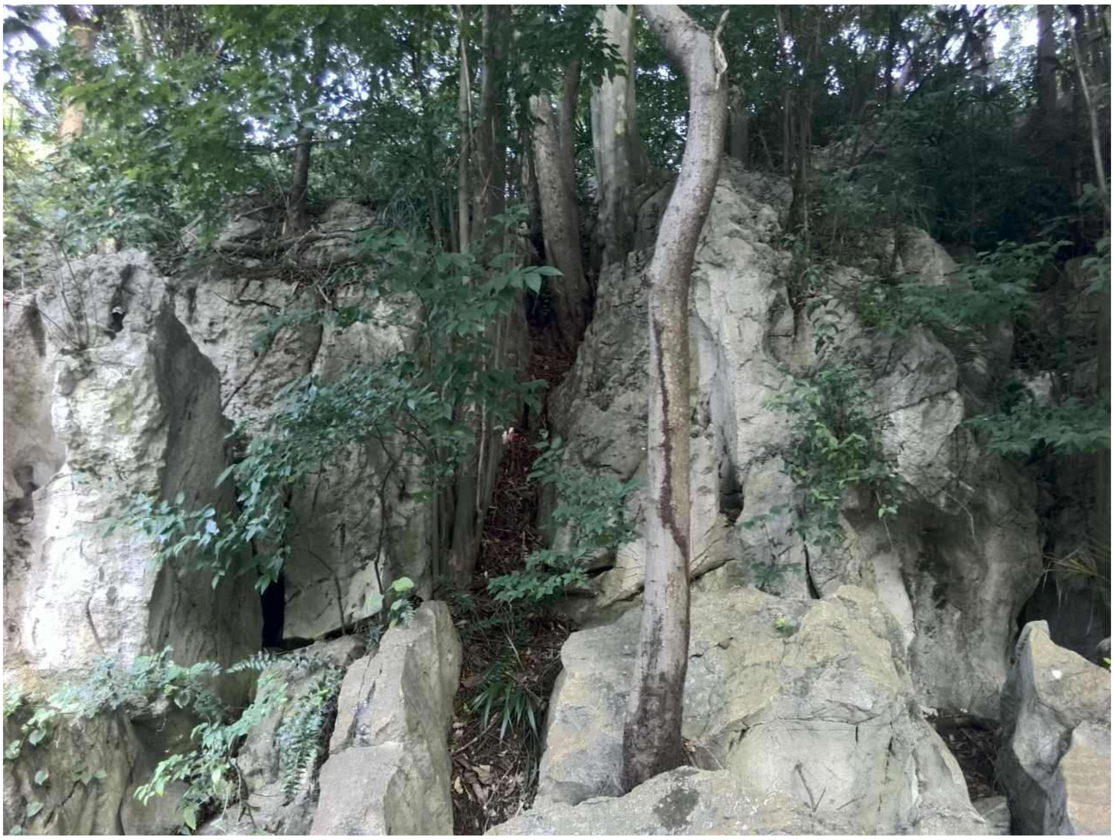

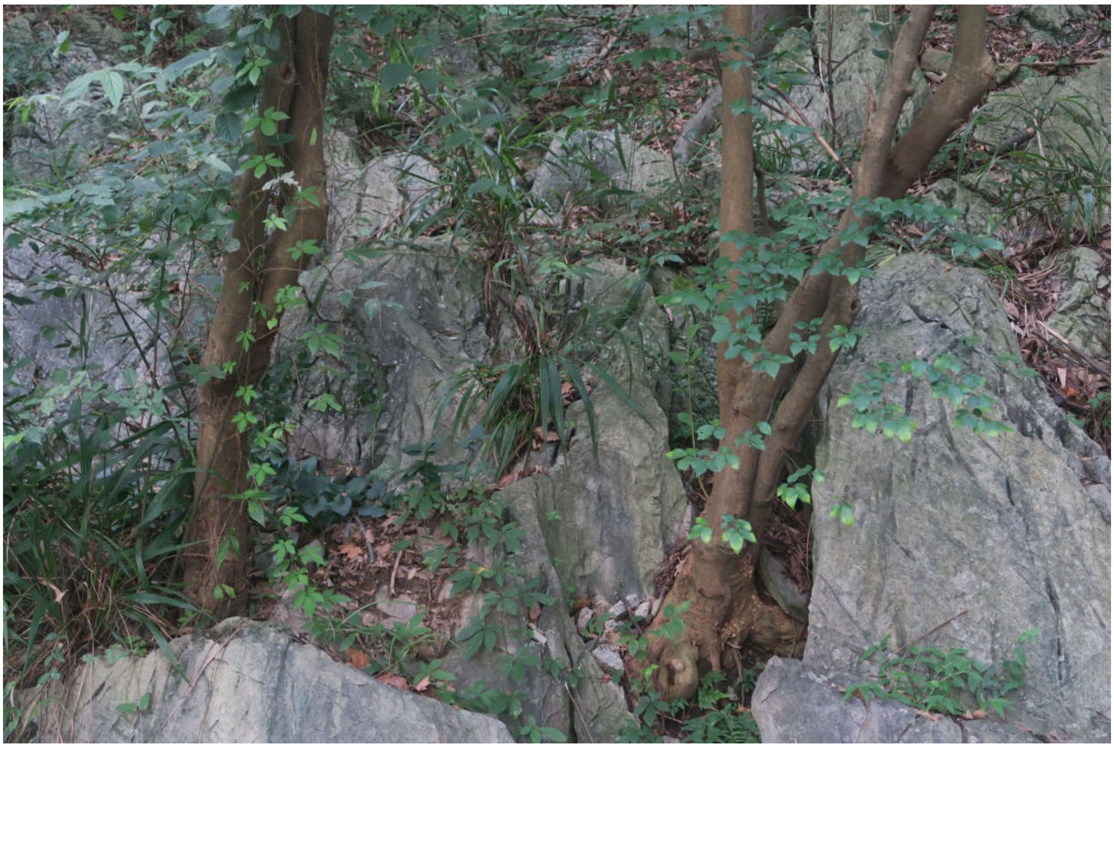

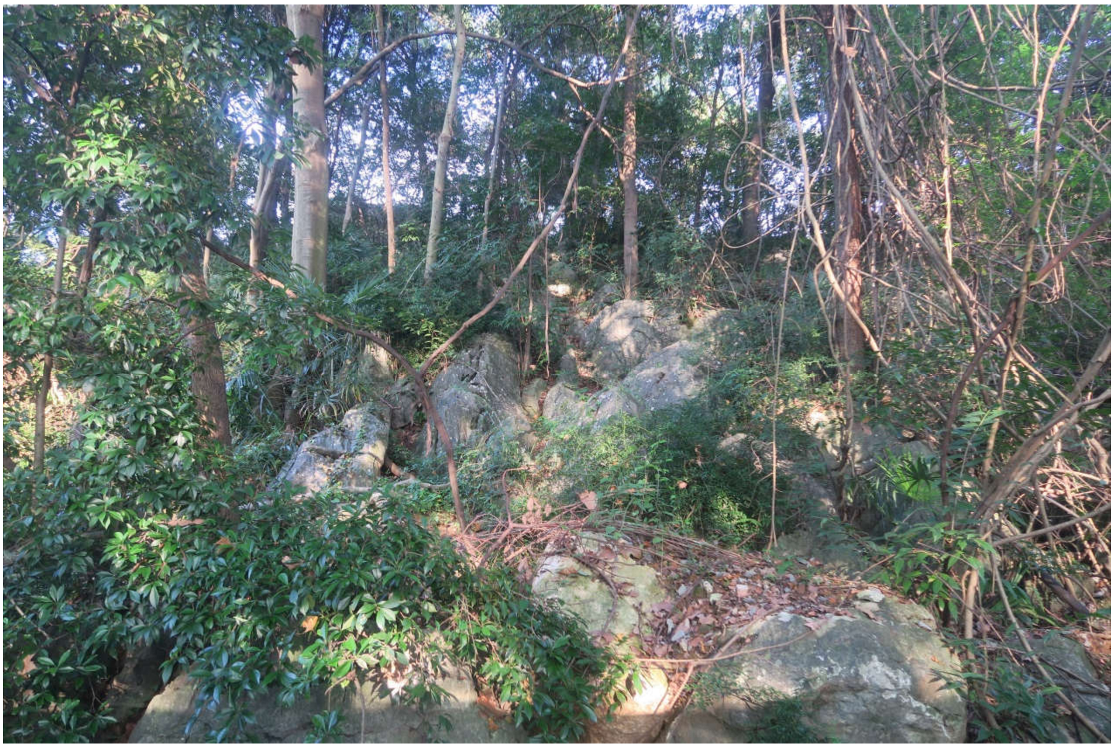

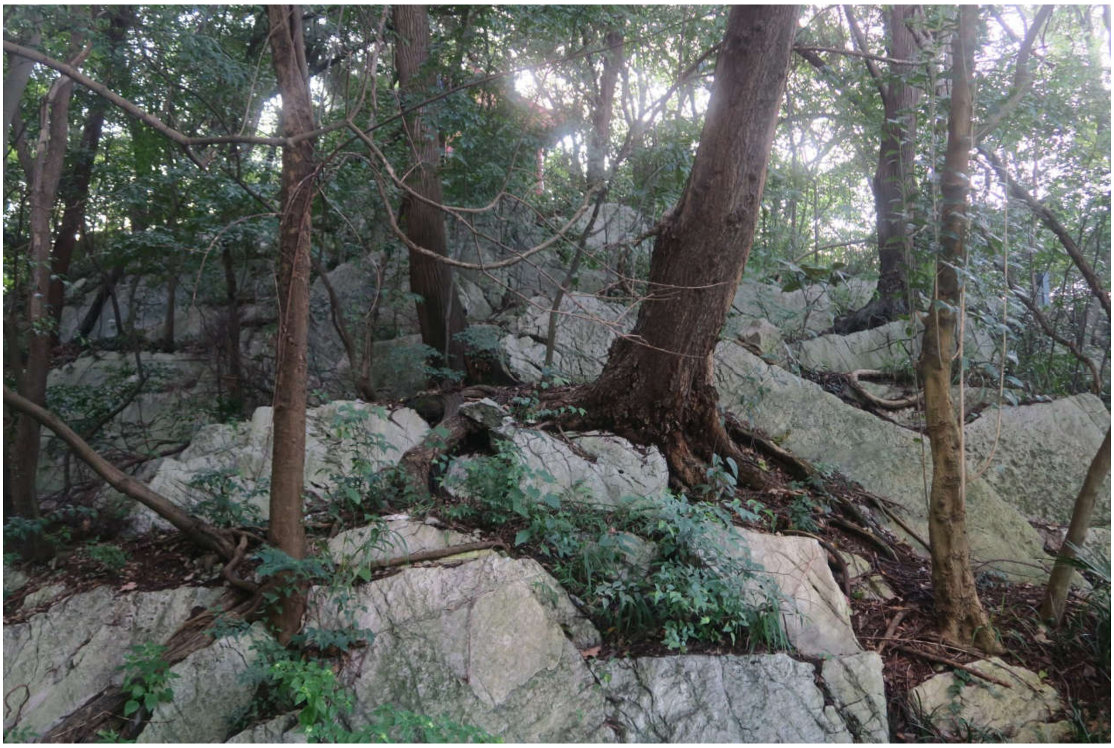

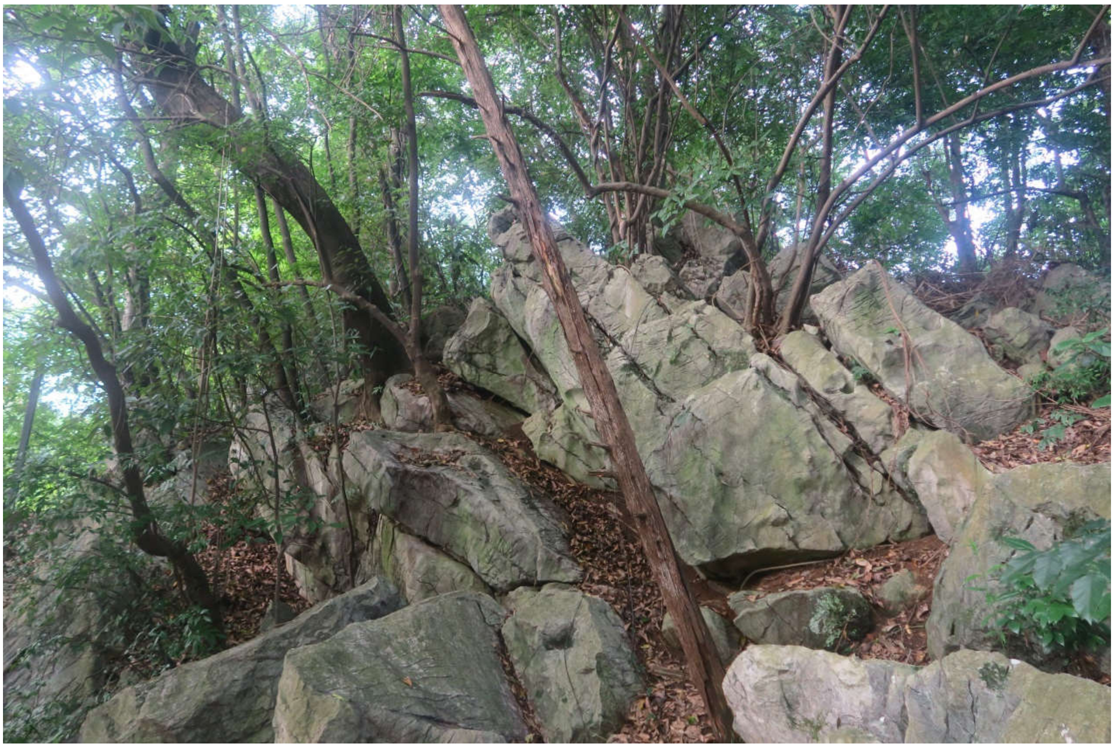

S5: Specific steps of GC-MS analysis for root exudates

We respectively sieved 40 g of the rhizosphere and non-rhizosphere soils of each species through a 40-mesh sieve and filled it in a 500 ml conical flask. We decanted 150 ml of dichloromethane into the conical flask. The conical flask was closed with a stopper and continuously oscillated for 1 hour. Subsequently, the mixture in the conical flask was extracted for 20 min using ultrasonic waves with power 1200 watts and filtrated. The residue was collected and placed into another 500 ml conical flask. Similarly, the root exudates of the residue were extracted. The filtrates from the two extractions were mixed, concentrated for 20 min with a rotary evaporator, and dissolved with 5 ml ether that was filtered through a 0.45 mm filter membrane. Finally, the liquid mixture was filled into a sterile centrifuge tube for GC-MS analysis. According to following references, the solutions that were gotten by above steps were generally considered to only include root exudates from soil samples.

The GC-MS analysis was performed using a HP 6890 gas chromatograph equipped with an Agilent MSD 5975C mass spectrometer. The GC-MS analysis was performed using a HP 6890 gas chromatograph equipped with an Agilent MSD 5975C mass spectrometer (Agilent Technologies) and chromatographic column (AB-5MS 5% phenyl-95% dimethylpolysiloxane; 30 m×0.25 mm×0.25 mm; elastic quartz capillary).

The temperature in the vaporization chamber was maintained at 250℃. Highly pure helium was used as the carrier gas, with a flow rate of 1.0 ml min-1. The inlet pressure was 7.62psi; the split ratio was 20:1. The solvent delay time was set to 1.5 min (Müller et al., 2008). The identification of the analogues of antibiotics and relative contents was conducted with a mass spectrometry data system. Specifically, the different peaks of total ion spectrum were first compared with the standard spectrum of the NIST05 and Wiley275 databases to determine the volatile constituents of the root exudates in soil. The peak area normalization method was then used to measure the relative mass fraction of the volatile constituents.

At last, the test results for a specific organic matter from soil samples in the non-rhizospheres of a plant species were deducted from the test results for the organic matter from soil samples in the rhizospheres of the plant species.

REFERENCES

1. Müller,A., Pietsch, B., Faccin, N., Schierle, J., Waysek, E.H. 2008. Method for the determination of lycopene in supplements and raw material by reversed-phase liquid chromatography: single-Laboratory Validation. Journal of AOAC International, 91, 1284–1297.
2. Leslie A. Weston, Ulrike Mathesius. 2014. Root Exudation: The Role of Secondary Metabolites, Their Localisation in Roots and Transport into the Rhizosphere. Edited by Asunción Morte, Ajit Varma. Root engineering. London: Springer. Doi: 10.1007/978-3-642-54276-3_11
3. Mukerji, K. G.， Manoharachary, C.， Chamola, B. P. 2002. Techniques in Mycorrhizal Studies. London: Springer.
4. Oburger, E, Dell‘mour, M, Hann, S., Wieshammer, G, Puschenreiter,M, Wenzel,W. 2012. Evaluation of a novel tool for sampling root exudates from soil-grown plants compared to conventional techniques. Environmental and Experimental Botany, 87:235-247.

S6: Results and processes of principal component analysis

Table A Standardized X based on Table 2 in main text

Species name	X1	X2	X3	X4	X5	X6	X7
I. yunnanensis	0.4942	0.4732	0.6222	0.6852	0.2636	0.3050	0.3405
Q. aliena	0.4173	0.2374	0.5881	0.6558	0.3810	0.1296	0.2094
L. lucidum	0.5295	0.1968	0.4524	0.0000	0.7033	0.4228	0.0000
I.chinensis	0.4557	0.3817	0.6189	0.6357	1.0000	0.4588	0.4088
C. glanduliferum	0.0000	0.2020	1.0000	0.9446	0.2359	0.4434	0.6386
A. kalkora (Roxb.)Prain	0.2628	0.0632	0.6172	0.6030	0.4262	0.4501	0.2667
C. sinensis	0.2908	0.2265	0.6970	0.6929	0.6358	0.1821	0.3688
C.tricuspidata	0.3960	0.3837	0.6880	0.5646	0.3410	0.4244	0.1669
B. papyifera	0.4549	0.4781	0.6378	0.4160	0.3263	0.4733	0.1944
Z.planispinum Sieb.et Zucc.	0.5295	0.3286	0.5049	1.0000	0.1483	0.3138	0.7020
T.asiatica	0.3845	0.4120	0.7096	0.5715	0.4368	0.1646	0.1295
R.martinii	0.5405	0.3177	0.4867	0.5233	0.4853	0.3606	0.2339
D.kaki var. silvestris	0.3266	0.1708	0.6380	0.6896	0.8078	0.1646	0.3251
R.chinensis	0.6815	0.5565	0.4869	0.9427	0.2567	0.4084	1.0000
I.corallina	0.3417	0.2695	0.6706	0.6198	0.5169	0.6111	0.4137
C. fargesii f. duclouxii	0.5007	0.3358	0.5525	0.6550	0.5271	0.2449	0.3245
D. cathayensis	0.4436	0.3886	0.6444	0.8051	0.3910	0.2356	0.4825
V.chinshanense	0.7657	0.8728	0.4824	0.5598	0.5355	0.1111	0.1731
E. pungens	0.3167	0.1593	0.6659	0.6589	0.7036	0.3776	0.3501
L.communis	0.4638	0.3647	0.5943	0.4285	0.5151	1.0000	0.5102
P.atalantioides	0.6161	0.5519	0.4826	0.5039	0.4524	0.2623	0.0812
L. cubeba	0.2774	0.1503	0.7304	0.3282	0.4201	0.4959	0.2015
M.microcarpa	1.0000	0.6246	0.0000	0.7763	0.8474	0.2814	0.5636
I. chinensis Hook.et Arn.var.oblonga	0.4009	0.4198	0.6610	0.4913	0.3318	0.4470	0.2322
I.memecylifolia	0.3465	0.2467	0.6584	0.6497	0.2968	0.3338	0.3576
L. formosana	0.1832	0.0995	0.8590	0.6396	0.5348	0.3971	0.4282
L. sinense	0.2709	0.3102	0.8202	0.6607	0.6404	0.4527	0.4147
M.philippensis	0.1652	0.1564	0.8806	0.5378	0.5184	0.4964	0.3124
L.glaber (Thunb.) Nakai	0.4331	0.3347	0.5041	0.6428	0.3347	0.3709	0.3661
A.mume	0.8098	1.0000	0.3470	0.8498	0.6639	0.0000	0.3154

C.mandarinorum	0.3535	0.2637	0.6408	0.7321	0.0000	0.2047	0.3585
C. glauca	0.5784	0.5378	0.4183	0.5681	0.3000	0.3369	0.2574
E.trichotoma (Lour.) Pierre var. pubescens Huang	0.1945	0.0000	0.6612	0.4775	0.6844	0.4347	0.2069
L.confinis Huang	0.5122	0.5675	0.6382	0.6127	0.4764	0.5566	0.4627
S.wilsoniana	0.4555	0.4510	0.6569	0.3315	0.5468	0.8292	0.4473
E. fargesii Dode	0.5343	0.3612	0.5298	0.6411	0.7649	0.2572	0.2700
V. canescens	0.2284	0.1004	0.6907	0.6684	0.8921	0.2371	0.2967
C.pubescens	0.4375	0.2695	0.5690	0.6396	0.7044	0.5144	0.2964
N. davidii	0.4496	0.3756	0.6102	0.7825	0.6422	0.2073	0.7668
P.longipes	0.4945	0.2637	0.5156	0.6256	0.7981	0.4902	0.4225
C. gracilis	0.5671	0.4867	0.5259	0.5537	0.3230	0.5123	0.3995
C.platycarpa	0.3643	0.3912	0.7222	0.7008	0.7193	0.5195	0.5844

Table B Characteristic vectors, eigenvalues, and variance contribution of components 1-4

Component	X1	X2	X3	X4	X5	X6	X7	Eigenvalues λ_i	Variance contribution (%)	Accumulation (%)
1	0.359	0.315	-0.326	0.106	0.022	-0.159	0.057	2.67	38.14	38.14
2	-0.109	-0.005	0.152	0.515	-0.212	-0.082	0.473	1.74	24.83	62.97
3	0.160	0.104	-0.098	-0.180	0.059	0.809	0.433	1.08	15.46	78.43
4	-0.047	-0.251	-0.086	0.188	0.925	-0.104	0.205	0.98	14.04	92.47

The characteristic vectors L_i calculated by Formula 6 and 7 corresponding to X1~X7 were given based on X, i.e., the rhizospher effects of plants on each index of SAE in Table 3. All L_i were <1 because the digits in Table 3 had been normalized. Aggregation status $X1$, aggregation degree $X2$ and dispersion ratio $X3$ were more explained by component 1 and 2 than other indices. Component 2 could explain much for dispersion coefficient $X4$. Organic matter $X5$ and particle fraction dimension $X6$ were primarily explained by component 4 and 3. Micro-aggregation fraction dimension was almost equally explained by component 2 and 3. Component 1, 2, 3 and 4 respectively took 38.14%, 24.83%，15.46% and 14.04% of variance contribution to the rhizosphere effects on SAE. The total variance contribution of the four

components reached 92.47%.

Table C Values of the principal factors Y_1-$Y4$

Species name	Y1	Y2	Y3	Y4	Y
I. yunnanensis	4.86	-1.34	1.78	1.91	2.24
Q. aliena	1.79	-4.42	1.55	13.29	1.83
L. lucidum	0.79	-34.49	12.32	31.56	-2.08
I.chinensis	4.42	-16.42	5.66	61.34	7.67
C. glanduliferum	-8.63	12.92	-7.08	4.70	-0.56
A. kalkora (Roxb.)Prain	-2.92	-6.21	0.80	17.92	-0.02
C. sinensis	-0.93	-6.51	1.15	34.13	3.24
C.tricuspidata	0.85	-6.08	2.44	7.28	0.23
B. papyifera	2.34	-11.56	5.09	3.49	-0.76
Z.planispinum Sieb.et Zucc.	7.58	10.78	-2.36	-1.68	5.37
T.asiatica	0.91	-7.41	2.73	14.76	1.08
R.martinii	5.09	-12.15	5.22	19.48	2.66
D.kaki var. silvestris	0.13	-10.34	2.35	48.56	5.04
R.chinensis	12.17	6.06	0.75	4.15	7.40
I.corallina	-0.14	-7.25	2.08	23.28	1.88
C. fargesii f. duclouxii	4.78	-7.72	3.36	24.17	4.13
D. cathayensis	4.03	0.87	0.29	14.41	4.13
V.chinshanense	14.59	-12.85	8.34	18.78	6.81
E. pungens	-0.97	-9.25	2.00	39.80	3.49
L.communis	2.31	-14.89	5.88	19.98	0.97
P.atalantioides	8.52	-12.61	6.56	14.43	3.41
L. cubeba	-5.48	-14.90	3.99	12.71	-3.66
M.microcarpa	24.47	-15.36	10.09	50.28	15.29
I. chinensis Hook.et Arn.var.oblonga	1.18	-8.66	3.57	5.40	-0.42
I.memecylifolia	-0.33	-2.28	0.53	6.21	0.29
L. formosana	-6.79	-4.89	-0.51	25.98	-0.26
L. sinense	-2.38	-6.77	1.24	32.90	2.40
M.philippensis	-7.50	-7.91	0.64	22.81	-1.65
L.glaber (Thunb.) Nakai	4.16	-4.67	2.35	8.87	2.20
A.mume	20.95	-6.30	6.82	32.15	12.97

C.mandarinorum	0.26	5.87	-1.83	-16.68	-1.15
C. glauca	9.02	-7.87	5.05	3.46	2.97
E.trichotoma (Lour.) Pierre var. pubescens Huang	-5.61	-14.71	2.81	37.57	-0.09
L.confinis Huang	5.76	-7.72	4.19	17.19	3.61
S.wilsoniana	1.56	-18.38	7.16	20.31	-0.01
T.glabrifolium	6.20	-12.88	5.14	42.91	6.47
V. canescens	-2.74	-11.84	1.97	55.64	4.46
C.pubescens	3.18	-11.14	3.80	38.83	4.85
N. davidii	4.80	-4.75	2.03	34.57	6.29
P.longipes	4.76	-13.93	5.03	46.36	6.10
C. gracilis	6.61	-7.96	4.65	5.16	2.15
C.platycarpa	1.67	-7.89	2.48	39.28	4.95

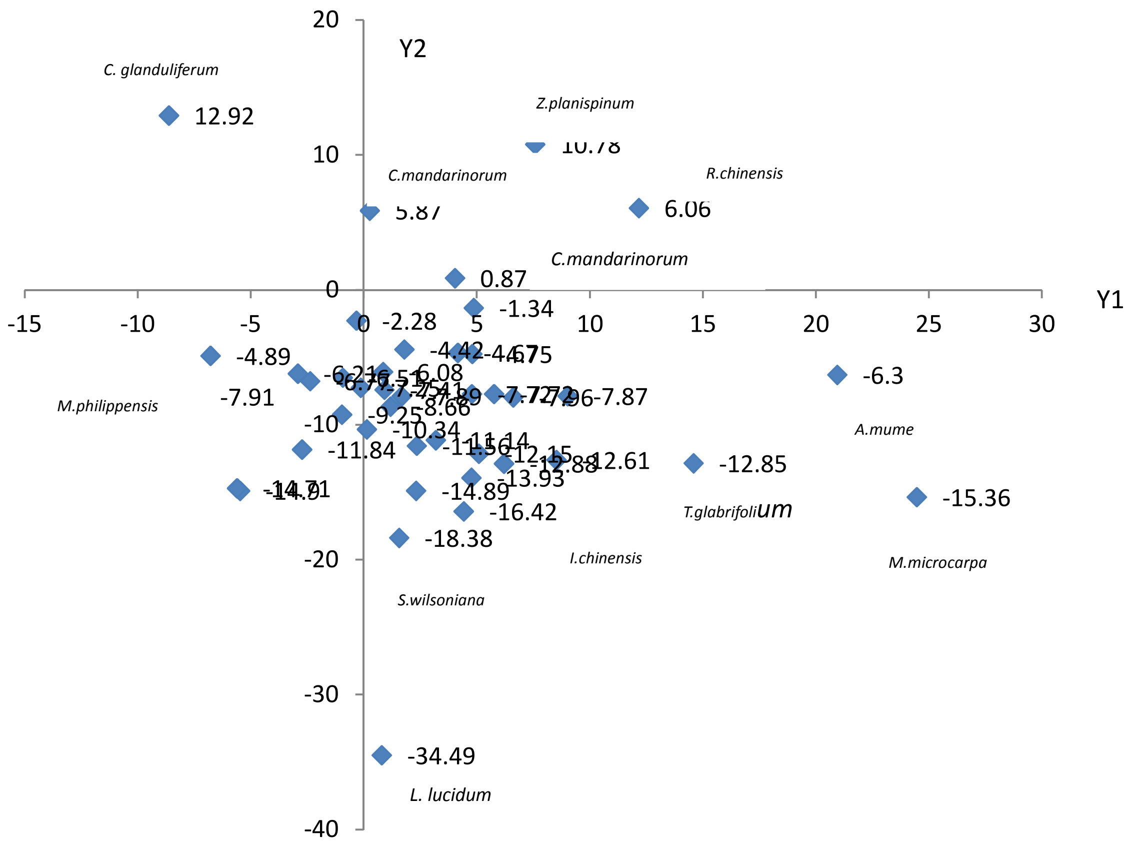

Figure A The two-dimensional plot of 7 variables by PCA

,

S7: Results and processes of redundancy analysis

$\hat{Y}=R(Q_kX) = R_1(Q_1X)+ R_2(Q_2X) +R_3(Q_3X) +R_4(Q_4X)$

Q_1X=0.359X1+0.315X2-0.326X3+0.106X4+0.022X5-0.159X6+0.057X7

Q_2X=-0.109X1-0.005X2+0.152X3+0.515X4-0.212X5-0.082X6+0.473X7

Q_3X=*0.159X1+0.104X2-0.098X3-0.180X4+0.059X5+0.809X6+0.433X7*

Q_4X=-0.047X1-0.251X2-0.086X3+0.188X4+0.925X5-0.104X6+0.205X7

R1=0.024; R2=-0.027; R3=-0.049; R4=-0.002;

There are 42 species studied and correspondingly the regression equation $\hat{Y}$ is obtained based on 42 groups of X1-X7 by regression analysis.

RI=0.839

Species name	$\hat{Y}$	ε
C. glanduliferum	0.24156	-0.00636
L. formosana	0.4021	0.013522
M.philippensis	0.41659	0.018855
C.mandarinorum	0.43078	0.016058
L. sinense	0.45911	0.019778
Z.planispinum Sieb.et Zucc.	0.46986	-0.05557
I.memecylifolia	0.47525	0.006502
A. kalkora (Roxb.)Prain	0.48255	-0.01335
C. sinensis	0.48884	0.04656
D. cathayensis	0.4902	0.019073
I.corallina	0.50391	-0.03512
V. canescens	0.50611	0.048547
E. pungens	0.50887	0.012297
E.trichotoma (Lour.) Pierre var. pubescens Huang	0.5097	0.006757
L. cubeba	0.50985	0.005458
C.tricuspidata	0.51044	0.035719
C.platycarpa	0.51303	-0.02172
T.asiatica	0.51862	0.09105
Q. aliena	0.51977	0.055694
I. yunnanensis	0.52178	0.024566
D.kaki var. silvestris	0.53003	0.051267
N. davidii	0.53484	-0.01807
I. chinensis Hook.et Arn.var.oblonga	0.53641	0.017092
R.chinensis	0.54341	-0.10063
L.glaber (Thunb.) Nakai	0.55304	-0.01661
L.confinis Huang	0.56699	-0.01933
C. fargesii f. duclouxii	0.57018	0.027603
B. papyifera	0.57189	0.017747
C.pubescens	0.57324	-0.00792
L.communis	0.58939	-0.12094
C. gracilis	0.59539	-0.03116
S.wilsoniana	0.60224	-0.07084
I.chinensis	0.6094	0.009785

P.longipes	0.61116	-0.02744
R.martinii	0.61547	0.007704
E.fargesii Dode	0.61888	0.041525
C. glauca	0.6346	0.004631
P.atalantioides	0.65465	0.060394
V.chinshanense	0.71089	0.097027
A.mume	0.73442	0.092508
L. lucidum	0.74339	0.015659
M.microcarpa	0.86703	-0.05857
